# Symmetry Breaking and Hydrogen Bonding in Phthalimide Compounds Enable Efficient Room‐Temperature Circularly Polarized Phosphorescence in Solution

**DOI:** 10.1002/anie.202515218

**Published:** 2025-10-09

**Authors:** Catherine Demangeat, Maxime Rémond, John M. Hudson, Emrys W. Evans, Denis Jacquemin, Ludovic Favereau

**Affiliations:** ^1^ Univ Rennes, CNRS, ISCR – UMR 6226 Rennes F‐35000 France; ^2^ Univ Angers, CNRS, MOLTECH‐Anjou, SFR MATRIX Angers F‐49000 France; ^3^ Department of Chemistry Swansea University Swansea SA2 8PP UK; ^4^ Centre for Integrative Semiconductor Materials Swansea SA1 8EN UK; ^5^ Nantes Université, CNRS, CEISAM UMR 6230 Nantes F‐44000 France; ^6^ Institut Universitaire de France (IUF) Paris F‐75005 France

**Keywords:** Organic chiral emitters, Room temperature circularly polarized phosphorescence, Spin orbit coupling, Symmetry breaking

## Abstract

The development of purely organic chiral room temperature phosphorescence (RTP) emitters is attracting more and more attention. However, the key parameters governing the polarized luminescence process remain difficult to predict and rationalize since the phosphorescence emission is rarely obtained in solution, hampering any structure‐relationship studies. To address this challenge, we report here the synthesis and chiroptical properties of a new family of metal‐free phosphorescent emitters based on phthalimide derivatives. Breaking symmetry of the phthalimide units and using intra‐ and intermolecular hydrogen bonding enable the obtention of circularly polarized (CP) RTP in solution with *g*_lum_ of up to 5 × 10^−3^. Interestingly, our investigations demonstrate the intricate role of hydrogen bonding interactions in modulating triplet state generation through the mixing of singlet and triplet states of different nature, i.e., n‐π* and π–π*, in the excited state, which is a crucial parameter for achieving intense CP‐RTP. These results bring additional molecular design guidelines to reach CP‐RTP in solution and additionally offer new insights into the subtle relationships between excited states of different spin multiplicity to reach higher CP phosphorescence intensity.

## Graphical abstract

Purely organic chiral room‐temperature phosphorescence (RTP) emitters are gaining increasing attention, but predicting the key factors governing polarized luminescence remains challenging, as RTP is rarely observed in solution. The crucial role of hydrogen bonding and symmetry breaking in modulating chiral triplet excited‐state interactions via n‐π* and π–π* state mixing is now highlighted, providing new molecular insights for enhancing circularly polarized RTP (*g*_lum_ up to 5 × 10^−3^).

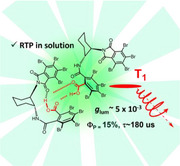

## Introduction

Room temperature phosphorescence (RTP) and related photophysical features of molecular materials related to the triplet excited states represent a fundamental topic for several research lines, including optoelectronics, non‐linear optics, photocatalysis, photodynamic therapy, and bioimaging.^[^ ^1^ ^]^ Historically, the radiative deexcitation of triplet excited states has usually been associated with heavy‐metal complexes, which can display efficient phosphorescence emission at RT due to a very efficient intersystem crossing (ISC) process promoted by the large spin‐orbit coupling (SOC) inherent to metallic centers.^[^ ^2^ ^]^

**Scheme 1:**
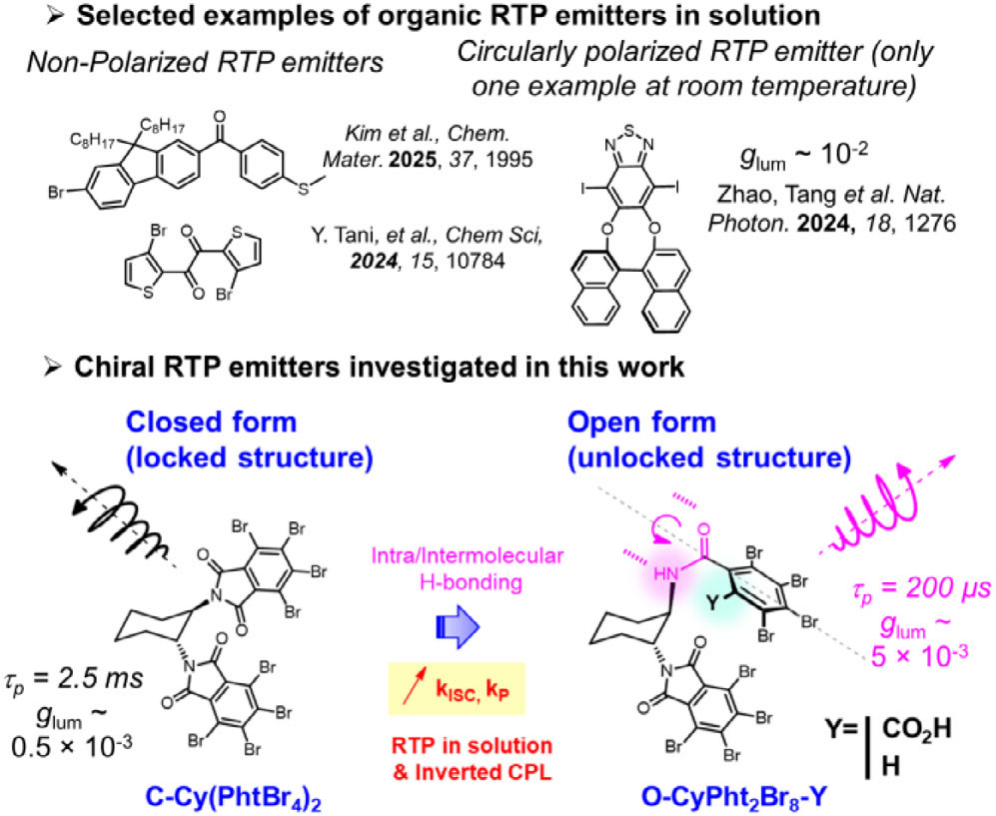
Chemical structures of selected phosphorescent emitters in fluidic medium (top) and of the phthalimide phosphorescent emitters investigated in this study (bottom), with an emphasis on the key findings regarding the obtained CP‐RTP in solution.

Recently, an increasing number of studies has described RTP from purely organic (metal‐free) molecules, offering an interesting direction to overcome environmental toxicity and cost issues encountered with organometallic species.^[^ ^3^, ^4^ ^]^ A general approach to promote RTP in organic compounds is to boost the ISC efficiency by introducing heavy halogen atoms. Alternatively, incorporating lone‐pair‐bearing heteroatoms, such as carbonyl groups, can also be a valuable strategy to enhance phosphorescence by promoting ISC between *n*‐π* and π–π* states of different spin symmetries, in accordance with El‐Sayed's rule.^[^ ^5^, ^6^, ^7^ ^]^ Along with these structural features, providing the phosphors a rigid environment is also crucial to restrict the internal molecular motions and to suppress the diffusion of triplet quenchers, such as molecular oxygen.^[^ ^8^, ^9^, ^10^ ^]^ Following these general guidelines, several types of organic compounds have been able to show RTP, with photoluminescence quantum yield (PLQY) up to 80% and lifetimes ranging from milliseconds to several seconds.^[^ ^11^ ^]^ However, in nearly all reported examples, phosphorescence has only been accessible in the solid state, where the joint influence of the surrounding matrix and intermolecular interactions on the excited‐state dynamics remains difficult to desconstruct.^[^ ^12^, ^13^, ^14^, ^15^, ^16^, ^17^, ^18^, ^19^ ^]^ This represents a key challenge in this field and highlights the still limited fundamental understanding of the RTP process in organic systems. To date, phosphorescence in fluid media has been observed for a very limited number of molecular designs, involving benzophenone^[^ ^20^, ^21^ ^]^ or 1,2‐diketones units^[^ ^22^, ^23^ ^]^ and more recently the thiadiazole moiety^[^ ^24^, ^25^, ^26^ ^]^ (Scheme 1). The limited examples have precluded the study of systematic structure–property relationship for this phenomenon in a non‐rigid environment.

While representing major issues in our understanding of the RTP of organic systems, such structure‐property relationships are also crucial for combining this emission process with other features, such as circular polarization. Recently, the development of CPL emitters involving purely organic emitters has attracted significant interest owing to the potential of CP‐light in a wide range of applications.^[^ ^27^, ^28^ ^]^ Therefore, merging this polarized emission with RTP has become a promising direction for obtaining CP‐RTP materials with metal‐free organic systems.^[^ ^29^, ^30^ ^]^ Indeed, the theoretically electric‐dipole‐forbidden nature of the triplet transition suggests a novel strategy to achieve high‐intensity polarized emission.^[^ ^31^, ^32^ ^]^ While this represents an interesting starting point, the limited examples of organic materials showing CP‐RTP in fluid media hampers a complete understanding of the structural and electronic factors (nature of the emitting transition, spin‐orbit coupling, and admixing between singlet and triplet excited states, to name a few) needed to obtain high intensity of CP‐RTP.^[^ ^33^ ^]^

In this framework, we report herein a study on CP‐RTP by exploring a family of chiral bisphthalimide derivatives^[^ ^34^, ^35^, ^36^, ^37^ ^]^ affording RTP ranging from green to orange colors with PLQY up to 15% (Scheme 1). In addition to the well‐known heavy atom effect,^[^ ^38^ ^]^ we show that symmetry breaking of the phthalimide units dramatically impacts the triplet excited state dynamics by boosting the SOC and therefore the ISC efficiency. Moreover, the introduction of both inter‐ and intramolecular hydrogen bonding interactions is a key requirement to enable more efficient and faster triplet‐state emission in the solid‐state, with lifetimes going from the milli‐ to the microsecond timescales. Interestingly, these two factors are crucial for obtaining rather high intensities of CP‐RTP, reaching *g*_lum_ value of up to 4 × 10^−3^ by notably impacting the energy of the emitting triplet state and the efficiency of its mixing with the singlet one. Finally, the obtained stabilized chiral aggregates additionally provide CP‐RTP in solution, with a promising *g*_lum_ of up to 5 × 10^−3^. These results provide one of the rare examples of CP‐RTP in fluid media for purely organic systems and offer new insights into the potential of “electric‐dipole forbidden” phosphorescence for achieving more intense CPL than classical spin‐allowed fluorescence of chiral emitters.

## Results and Discussion

### Synthesis and Characterizations

**Figure 1:**
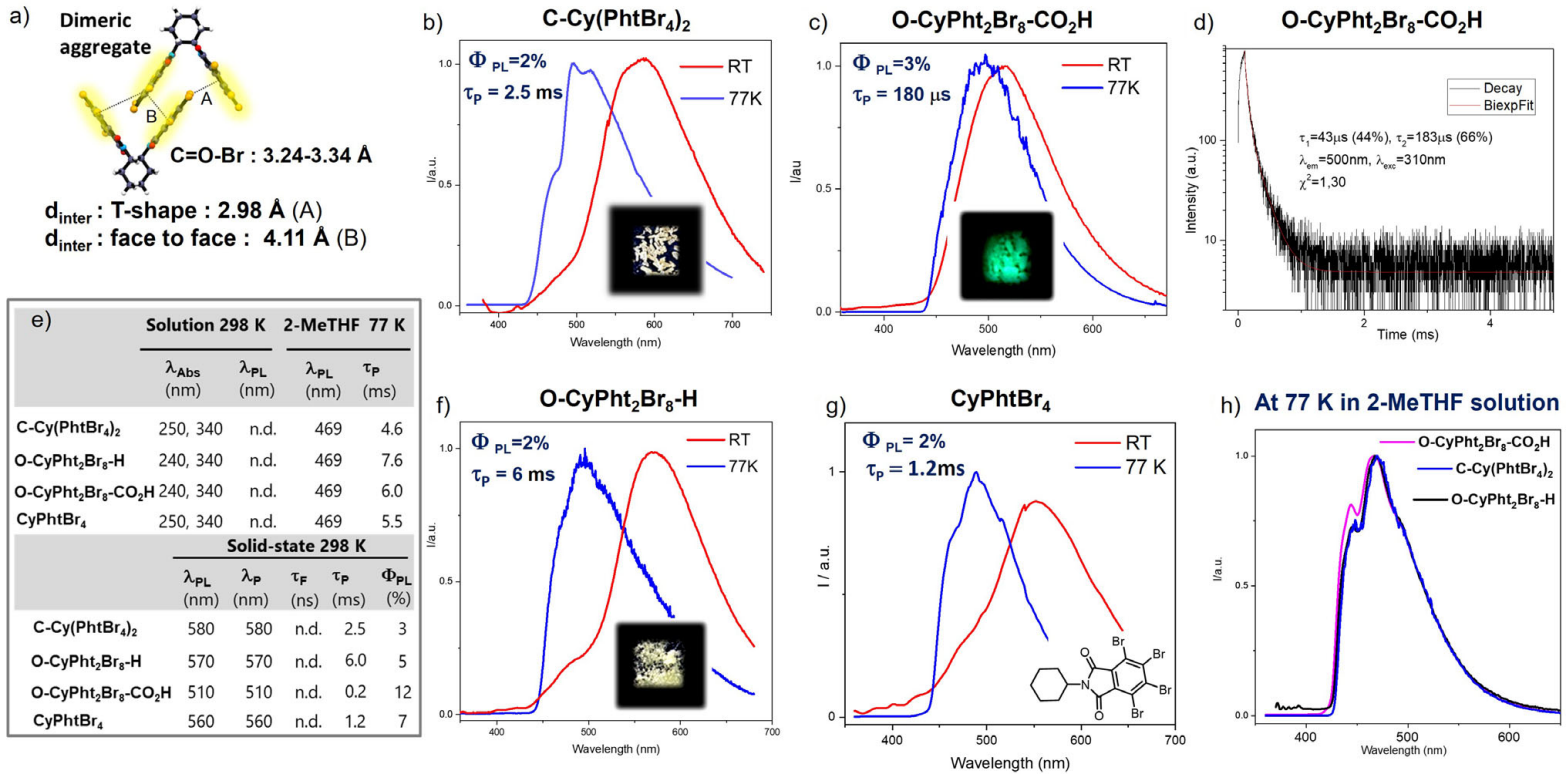
a) Main features extracted from X‐ray single crystal data obtained for **C‐Cy(PhtBr_4_)_2_** with intermolecular centroid–centroid distances between the phthalimides dimers; PL spectra recorded at 298 K (red solid line) and at 77 K (blue) of the solids b) **C‐Cy(PhtBr_4_)_2,_** c) **O‐CyPht_2_Br_8_‐CO_2_H**, f) **O‐CyPht_2_Br_8_‐H,** g) **CyPhtBr_4_** excited at λ_exc_ = 340 nm, with pictures of the luminescence obtained in the solid state at 298 K under UV lamp at λ = 365 nm; d) Lifetime decay profiles of phosphorescence with the corresponding values of lifetimes (τ) for **O‐CyPht_2_Br_8_‐ CO_2_H** at 510 nm under 312 nm excitation at 298 K; e) Photophysical data of the compounds recorded in chloroform at 298 K, in 2‐methyltetrahydrofuran at 77 K, and in solid‐state (crystals) at 298 K; h) PL spectra at 77 K of the different phthalimide derivatives diluted in 2‐MeTHF, under 340 nm excitation.

The synthesis of the compounds has been adapted from the one reported for fluorinated phthalimide derivatives.^[^ ^39^ ^]^ In contrast to what we experienced, mixing one equivalent of the enantiopure (*RR*)‐ or (*SS*)‐cyclohexane‐1,2‐diamine with the tetrabromo phthalic anhydride in refluxing acetic acid did not afford the symmetric bis‐phthalimide system directly, but rather an intermediate in which one phthalimide unit was still open with a remaining carboxylic group, **O‐CyPht_2_Br_8_‐CO_2_H** (Scheme S1). Heating this compound in DMF at 140 °C resulted in a quantitative decarboxylation reaction, leading to another derivative, **O‐CyPht_2_Br_8_‐H**. Finally, the symmetric closed form **C‐Cy(PhtBr_4_)_2_**, was obtained by performing the condensation reaction in toluene under basic conditions, with a satisfactory yield (up to 77%, see Supporting Information for details). These enantiopure derivatives were fully characterized by both NMR and mass spectrometry, displaying characteristic signals of both phthalimide (for O‐CyPht2Br8‐H) and cyclohexane units in the aromatic and aliphatic parts of the ^1^H spectrum, respectively. For instance, a characteristic doublet peak at 8.59 ppm can be observed for the amide proton on the nitrogen atom for the open products **O‐CyPht_2_Br_8_‐H** and **O‐CyPht_2_Br_8_‐CO_2_H**, as well as two non‐equivalent multiplet signals for the protons in the alpha position of the amide groups (see the Supporting Information for details). Surprisingly, irradiation of the obtained solids by UV light at λ = 365 nm (benchtop UV lamp) affords different emission colors, ranging from green to yellow, despite their rather similar chemical structure (Figure 1b,c,f). This behavior prompted us to further explore the obtained emission, as it opens up a rare opportunity for potentially rationalizing electronic and steric molecular factors governing CP‐RTP.

### Photophysical and chiroptical properties: preliminar investigations at the molecular level and conformational effectest(ce

The optical and photophysical properties of these chiral compounds were firstly investigated in diluted chloroform solution, resulting in similar UV–vis absorption signature for the open phthalimide **O‐CyPht_2_Br_8_‐H** and **‐CO_2_H** compounds and their closed **C‐Cy(PhtBr_4_)_2_** counterpart. The UV‐vis spectra show an intense absorption band at ca. 260 nm (ε ∼ 5.0 × 10^4^ M^−1^ cm^−1^) and smaller, broader ones between 300 and 370 nm (ε = 2.0 × 10^3^ M^−1^ cm^−1^), respectively, assigned to π‐π* and n‐π* transitions (Figure S16). Electronic circular dichroism (ECD) spectra of these compounds afford more insights regarding the impact of the additional structural freedom in **O‐CyPht_2_Br_8_‐H** and **‐CO_2_H**, in comparison to **C‐Cy(PhtBr_4_)_2_**. Indeed, while the three compounds show expected mirror‐image signals for these transitions in the spectra, the ECD response of both **O‐CyPht_2_Br_8_‐H** and **O‐CyPht_2_Br_8_‐CO_2_H** appears to be less intense and notably blueshifted in the 300–350 nm region. This can be explained by the less favored chiral excitonic coupling between the perpendicular in‐plane electronic dipoles present in each phthalimide unit, which are non‐degenerate in both open systems, especially for the π–π* transitions around 250 nm.^[^ ^40^, ^41^, ^42^ ^]^

Further insights regarding ground and excited states properties were obtained by theoretical calculations (see Figures S21–S30 for details). In the symmetric derivative **C‐Cy(PhtBr_4_)_2_**, only one conformer could be identified, whereas in both **O‐CyPht_2_Br_8_‐H** and **O‐CyPht_2_Br_8_‐CO_2_H,** various conformers close in energy were detected by DFT, all presenting similar UV‐vis features but rather different ECD signatures. The TD‐DFT optical spectra reasonably reproduce the experimental ones (Figures S24–S26), including, importantly, the ECD intensity decrease upon opening one imide ring and then removing the carboxylic group. Indeed, for **O‐CyPht_2_Br_8_‐H**, the ECD responses at both ∼250 and ∼320 nm of the most stable conformer predicted by TD‐DFT are markedly smaller than in **C‐Cy(PhtBr_4_)_2_** (see Figure S27 and discussion below). Interestingly, an intramolecular hydrogen bond can be observed between the amide group of the open phthalimide unit and one carbonyl group of the opposite closed phthalimide one for calculated structures of both **O‐CyPht_2_Br_8_‐H** and **O‐CyPht_2_Br_8_‐CO_2_H**, presumably participating in stabilizing one main conformer. In the case of **O‐CyPht_2_Br_8_‐CO_2_H**, the carboxylic group points outside of the molecule and can likely be engaged in further hydrogen bonding to form dimeric structures, as is usually observed for compounds having acid groups. This might significantly impact the intermolecular arrangement of such type of compounds and consequently their photophysical properties obtained in both solid‐state and solution (vide infra).

**Figure 2:**
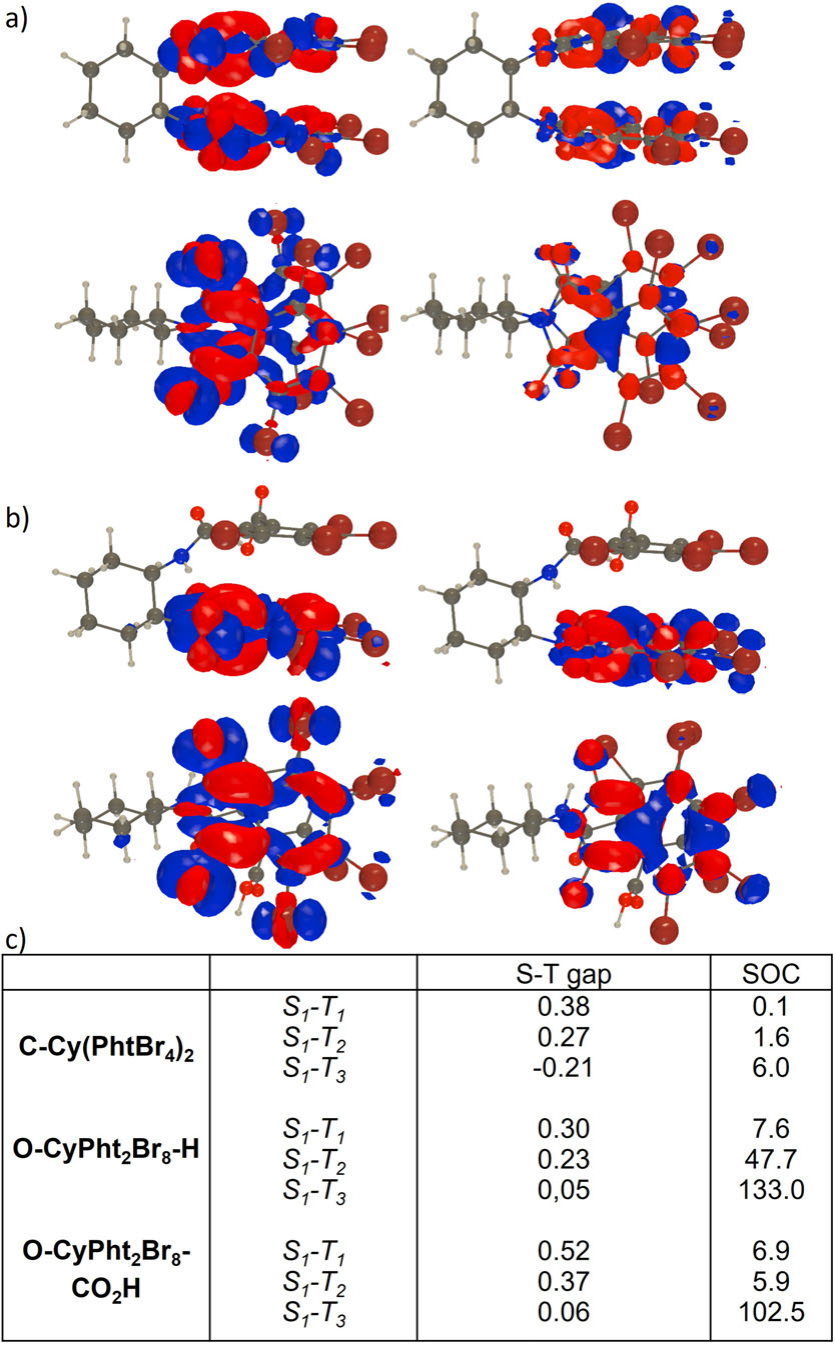
Electron density difference plot (two different views of the structure) of the lowest singlet (left) and triplet (right) excited states determined at TD‐CAM‐B3LYP/6‐311++G(d,p) level for: a) **C‐Cy(PhtBr_4_)_2_**; and b) the most stable conformer of **O‐CyPht_2_Br_8_‐CO_2_H**. The blue (red) regions indicate decrease (increase) of electron density upon absorption. Contour threshold: 0.001 au. c) SOC (cm^−1^) computed between the lowest *S_1_* state and the three lowest triplet states on the optimal *S_1_* geometry, with the associated S‐T gaps in eV, a negative value indicating a higher‐lying triplet.

In solution, the compounds are poorly emissive owing to an efficient ISC process from the singlet to the triplet excited states of the molecule (PLQY, ϕ < 0.01), as already described for phthalimide derivatives.^[^ ^43^ ^]^ Theoretical calculations performed on the optimized S_1_ geometry indeed reveal rather small S‐T gaps and large SOCs (Figure 2), with, interestingly, significantly larger SOCs in both **O‐CyPht_2_Br_8_‐H** and **O‐CyPht_2_Br_8_‐CO_2_H** than in **C‐Cy(PhtBr_4_)_2_**. The symmetry breaking in the two former “open” compounds aids the mixing of excited‐state characters, in turn, promoting more efficient ISC. From the analysis of the topology of the lowest singlet (S_1_) and triplet (T_1_) excited states (see electron density difference plots in Figures 2 and S28–S30), it turns out that the former shows a dominant n‐π* character, whereas the latter has a π–π* character. Interestingly, for **O‐CyPht_2_Br_8_‐H**, one also notices a small charge‐transfer contribution from one phthalimide unit to the other. For both open structures, the SOCs are particularly strong with the third triplet (S_1_ ‐> T3, Figure 2c), which has a π–π* character as well as a charge‐transfer character, with significant contributions from some of the bromine atoms. (see Figures S31–S33).

We then investigated the photophysical properties of the molecules at 298 K in the solid state. As shown in Figure 1b,f, the photoluminescence (PL) spectra of both **C‐Cy(PhtBr_4_)_2_** and **O‐CyPht_2_Br_8_‐H** display similar orange emission profiles characterized by one major emission peak at 580 and 570 nm, respectively, associated with PL quantum yield (QY) of ca. 5%–10% under inert conditions (Figure S3). For these two compounds, time‐resolved photoluminescence measurements confirmed the phosphorescent nature of this emission, with corresponding lifetimes of 2.5 and 6.0 ms for **C‐Cy(PhtBr_4_)_2_** and **O‐CyPht_2_Br_8_‐H**, respectively (Figures 1e and S1). In comparison, an intense blueshifted green PL spectrum is observed for **O‐CyPht_2_Br_8_‐CO_2_H** with an emission maximum at 510 nm (Figure 1c). The associated PLQY is ca. 15%, i.e., much more intense than for the closed system (Figure S3), with also a measured lifetime one order of magnitude smaller than the two other systems (τ_P_∼180 µs, Figure 1d). The emission differences between the various systems observed at 298 K are also preserved when measuring the solid‐state phosphorescence at 77 K (Figure 1b,c,f). While the emission maxima of both **C‐Cy(PhtBr_4_)_2_** and **O‐CyPht_2_Br_8_‐H** are blueshifted from 570–580 nm to 490–510 nm, the emission peak of **O‐CyPht_2_Br_8_‐CO_2_H** remains localized around 500 nm at 77 K. The only impact of the temperature for the latter one is the change of lifetime, 1.2 ms, one order of magnitude higher than at 298 K. This trend is also present for the two other solids, but to a significantly smaller extent (τ = 2.6 and 13.3 ms for **C‐Cy(PhtBr_4_)_2_** and **O‐CyPht_2_Br_8_‐H**, respectively, Figure S1).

The solid‐state spectra measured at 77 K for both **C‐Cy(PhtBr_4_)_2_** and **O‐CyPht_2_Br_8_‐H** are reminiscent of the ones recorded in dilute 2‐MeTHF solution also at 77 K (see Figures 1e,h and S9). Indeed, in these conditions and under 340 nm excitation, steady‐state and delayed emission exhibit one single structured emission band centered at 470 nm for all compounds, including **O‐CyPht_2_Br_8_‐CO_2_H**. This emission arises from the π‐π* triplet state of the phthalimide unit, as already described for **C‐Cy(PhtBr_4_)_2_**,^[^ ^38^ ^]^ associated with a lifetime of ca. 7.0 ms regardless of the considered compound (Figure S9). The temperature and concentration dependency of the phosphorescence emission (Figure S8) supports the important role of intermolecular interactions behind the phosphorescence emission behavior in the neat solid state. Despite numerous attempts, no crystal could be obtained for **O‐CyPht_2_Br_8_‐H** nor **O‐CyPht_2_Br_8_‐CO_2_H**, likely due to their poor solubilities in common organic solvents. However, the X‐ray analysis of **C‐Cy(PhtBr_4_)_2_** is helpful to rationalize these observations since it reveals a strong tendency of the phthalimide units to arrange themselves following a dimeric intermolecular association (Figure 1a and Table S1). The similar emission responses recorded at 570–580 nm and millisecond lifetime range for both **C‐Cy(PhtBr_4_)_2_** and **O‐CyPht_2_Br_8_‐H** strongly hint a similar excited‐state dimeric interaction also in the latter compound. This hypothesis is reinforced by the similar phosphorescence obtained in the solid state for the reference monophthalimide compound, **CyPhtBr_4_** (Figures 1g and S2). For both **C‐Cy(PhtBr_4_)_2_** and **O‐CyPht_2_Br_8_‐H**, one can clearly distinguish the phosphorescence process at a higher energy (∼480 nm) resulting from a triplet excited state localized on a phthalimide unit (monomer state) and the second process observed here at 570–580 nm, likely resulting from intermolecular interactions; the latter process requires an activation energy to allow delocalization of the triplet over several structures.^[^ ^3^ ^]^ Conversely, the absence of such phosphorescence at 570 nm for **O‐CyPht_2_Br_8_‐CO_2_H** excludes that type of dimeric interaction, therefore indicating that the carboxylic group tunes the packing, hence affecting the localization of the triplet exciton and its resulting radiative deexcitation in the solid‐state at 510 nm.

**Figure 3:**
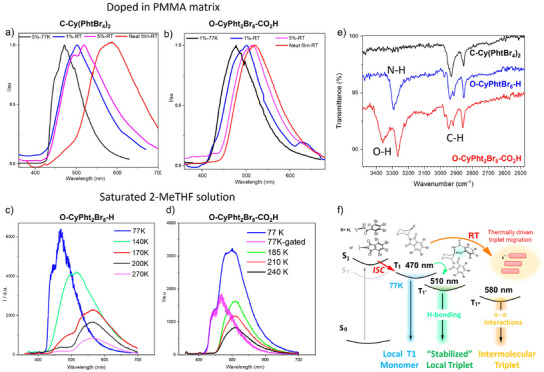
a) Photoluminescence spectra of **C‐Cy(PhtBr_4_)_2_** dispersed in PMMA matrix with different doping rates and temperatures under 340 nm excitation; b) Photoluminescence spectra of **O‐CyPht_2_Br_8_‐CO_2_H** dispersed in PMMA matrix with different doping rates and temperatures under 340 nm excitation; c) Steady state PL spectra of **O‐CyPht_2_Br_8_‐H** at different temperatures from 77 to 270 K in saturated 2‐MeTHF solution under 340 nm excitation; d) Steady state and 1 ms‐gated PL spectra of **O‐CyPht_2_Br_8_‐CO_2_H** at different temperatures from 77 to 240 K in saturated 2‐MeTHF under 340 nm excitation; e) FTIR analysis of the solid powders and f) schematic illustration of the photophysical events occurring for the investigated compounds.

Indeed, while more freedom is given to the molecular structures in both **O‐CyPht_2_Br_8_‐CO_2_H** and **O‐CyPht_2_Br_8_‐H**, and therefore more conformational flexibility, only **O‐CyPht_2_Br_8_‐CO_2_H** shows a different phosphorescent emission. It looks plausible that the hydrogen bonding anchor offered by the carboxylic group plays a key role here. To confirm this hypothesis, we measured the IR spectra of the compounds in the solid‐state, see Figures 3e, S19, and S20). As can be seen in both **O‐CyPht_2_Br_8_‐CO_2_H** and **O‐CyPht_2_Br_8_‐H** the N–H stretching vibration appears in the 3290–3240 cm^−1^ range, while the amide carbonyl stretch stands between 1657 and 1640 cm^−1^ (and the amide II falls between 1560 and 1540 cm^−1^). These wavenumber ranges are characteristic of H‐bonding since NH stretch vibrations of free amide functionalities are typically found at frequencies about 200 cm^−1^ higher (∼3450 cm^−1^).^[^ ^44^, ^45^, ^46^ ^]^ For **O‐CyPht_2_Br_8_‐CO_2_H**, this NH signal is further redshifted by 30 cm^−1^, indicating a stronger hydrogen bonding. In addition, the O─H stretching vibration of the carboxylic group is recorded at 3360 cm^−1^ and appears well‐defined, suggesting its implication in hydrogen interactions, presumably through the formation of carboxylic acid dimers in the solid state. This is also probed by the acid carbonyl vibration peak found at 1732 cm^−1^, which is characteristic of H‐bonded carbonyl stretch in dimeric carboxylic acid derivatives. Such additional hydrogen bond likely precludes efficient intermolecular phthalimide–phthalimide interaction in the excited‐state and leads to different triplet excited‐state dynamics.

To gain further insights into the impact of these secondary interactions on the molecular assembly and associated triplet exciton dynamics, the photophysical properties of **C‐Cy(PhtBr_4_)_2_**, **O‐CyPht_2_Br_8_‐H**, and **O‐CyPht_2_Br_8_‐CO_2_H** were investigated in PMMA matrix, with 1wt% and 5 wt% doping ratios. As shown in Figure 3a,b, the steady‐state PL spectra of the drop‐casted film of both **C‐Cy(PhtBr_4_)_2_** and **O‐CyPht_2_Br_8_‐H** displayed a similar continuous blueshifted evolution from 570 to 500 nm at RT upon decreasing their concentration from 5 to 1 wt% (see also Figures S5–S7). The same observation can be made for the reference **CyPhtBr_4_** (Figure S5), thus confirming the dimeric nature of the emissive excited state obtained at 570–580 nm in the pure solid‐state for these three systems. Indeed, recording the emission spectrum of the PMMA films at 77 K afforded an emission response getting very close to that of the monomeric triplet, recorded in diluted 2‐MeTHF for these three compounds. In contrast, these changes are less obvious for **O‐CyPht_2_Br_8_‐CO_2_H**, as the spectral shape of the 5wt% PMMA? film remains very similar to the one of the pure solid (Figure 3b). Decreasing further this ratio to 1wt% slightly shifts the emission from 510 to 497 nm, the latter value being highly similar to the one of the other derivatives, though the spectrum remains broader than those of **C‐Cy(PhtBr_4_)_2_** and **O‐CyPht_2_Br_8_‐H** at 77 K. This indicates the presence of two emissive states: the one observed in the pure solid at 510 nm and the other one localized to one phthalimide unit at 480 nm (Figure S7). Additional evidences on this aspect were obtained by recording steady‐state and delayed emission spectra of highly concentrated solution of **O‐CyPht_2_Br_8_‐CO_2_H** in 2‐MeTHF at 77 K (Figures 3d and S8–S11).

As shown in Figure 3d, the obtained steady‐state PL spectrum of **O‐CyPht_2_Br_8_‐CO_2_H** displays a very similar signature to the one obtained in PMMA matrix, with, however, a distinguishable shoulder at 450 nm. Using a time gate of 1 ms allows us to isolate only a long‐lived high‐energy emission component at 470 nm with identical features to the one? in dilute 2‐MeTHF frozen solution (Figure S8), which therefore emits concomitantly with the more intense and faster one at 500 nm in these conditions. Additional excitation spectra of this 500 nm emission also support the formation of a new ground state molecular conformation upon hydrogen bonding (Figure S11) and highlight its role in the excited state associated with the phosphorescence of **O‐CyPht_2_Br_8_‐CO_2_H**. In sharp contrast, the steady‐state PL spectra of concentrated solution of **C‐Cy(PhtBr_4_)_2,_ CyPhtBr_4_**, and **O‐CyPht_2_Br_8_‐H** in 2‐MeTHF at 77 K exhibit identical emission spectra to those in dilute frozen solutions, with a structured emission peak at 470 nm (Figures 3c and S10). Additionally, the PL spectra of **C‐Cy(PhtBr_4_)_2,_ CyPhtBr_4_**, and **O‐CyPht_2_Br_8_‐H** shift progressively to 570 nm with increasing temperature, while for **O‐CyPht_2_Br_8_‐CO_2_H,** the signal stays centered at 500 nm, with an intensity continuously declining until it reaches RT (Figure 3c,d). For arylimide derivatives, it has been reported that hydrogen bonding could occur in the excited state of the molecule, involving notably the carbonyl group.^[^ ^47^ ^]^ This interaction can impact both the luminescence maximum and lifetime by inducing an energy increase of the n‐π* excited‐state and a slight stabilization of the π–π* one. Since a hydrogen bond involving a carbonyl group of **O‐CyPht_2_Br_8_‐CO_2_H** is already present in the ground state, the latter is likely to be present in the excited‐state, therefore redshifting the RTP and enhancing its radiative efficiency, in comparison to the two other derivatives, **C‐Cy(PhtBr_4_)_2_** and **O‐CyPht_2_Br_8_‐H**. In addition, the phosphorescence observed for the two latter at 570–580 nm results from a delocalized triplet excited‐state over several phthalimide units because of intermolecular interactions. Accordingly, their corresponding radiative deexcitation likely involves a more complex picture, which may be characterized by a lower oscillator strength and/or a smaller radiative rate. A schematic illustration of the photophysical solid‐state behavior of **C‐Cy(PhtBr_4_)_2,_ O‐CyPht_2_Br_8_‐H**, and **O‐CyPht_2_Br_8_‐CO_2_H** is depicted in Figure 3f to highlight the main aspects observed in terms of phosphorescence in function of both the structural differences between the investigated compounds and the temperature.

### Photophysical and Chiroptical Properties in Solution

During our investigations, we noticed that all compounds displayed phosphorescence also in fluid CHCl_3_ solution ([] ∼10^−4^ M) when excited at 340 nm at room temperature. **C‐Cy(PhtBr_4_)_2,_ O‐CyPht_2_Br_8_‐H**, and **O‐CyPht_2_Br_8_‐CO_2_H** exhibit similar PL spectra as those recorded for the solid compounds, with a major emission peak at 560, 570, and 510 nm, respectively (Figure S12). These phosphorescence emissions were then associated with small molecular aggregates in the range of 300 nm from dynamic light scattering experiments. The measured luminescence liftetime values are 5 µs, 5 ms, and 150 µs for **C‐Cy(PhtBr_4_)_2_**, **O‐CyPht_2_Br_8_‐H**, and **O‐CyPht_2_Br_8_‐CO_2_H**, respectively (Figure S12), which are nearly the same as the ones measured in the solid state, except for **C‐Cy(PhtBr_4_)_2._** For the latter, the recorded shortened lifetime reflects the poor ability of the molecular aggregates to yield phosphorescence in solution in comparison to **O‐CyPht_2_Br_8_‐H** and **O‐CyPht_2_Br_8_‐CO_2_H**. While the three compounds show intermolecular π‐π interactions, it seems that the presence of additional secondary interactions such as the hydrogen ones and/or an enhanced flexibility helps promote RTP in fluid media. Intriguingly, the phosphorescence of **O‐CyPht_2_Br_8_‐CO_2_H** in solution evolves with time and is ultimately redshifted to 550 nm after 30 min in CHCl_3_ solution (298 K), the new signal having much weaker intensity and broader profile (Figure S13). At the same time, the lifetime drops from 150 µs to 4 µs, indicative that the H‐bonded aggregate initially formed in the solid state evolves towards a new state with similar characteristics to that recorded for the closed **C‐Cy(PhtBr_4_)_2_** derivative.

The role of aggregates in solution is further supported by the clear dependence of the RTP intensity on the concentration of the compounds, with PL peak hardly observed in dilute conditions (µM, Figures S14 and S15). We also performed picosecond transient absorption (TA) spectroscopy studies of phthalimide derivatives in chloroform. Ongoing from dilute (20 µM) to more concentrated samples (100 µM, 1 mM) of **CyPhtBr_4_**, we find the same initial excited‐state dynamics, which we attribute to monomeric ISC (300 ps), followed by secondary evolution to a broad 380–500 nm excited‐state absorption feature assigned to the intermolecular triplet state (see Supporting Information for further details). This picture of monomeric to intermolecular triplet formation is also consistent with our studies on **C‐Cy(PhtBr_4_)_2_** (Figures S34 and S35), although unfortunately we could not conduct the same TA experiments on open compounds due to low solubility and film quality despite significant efforts.

**Figure 4:**
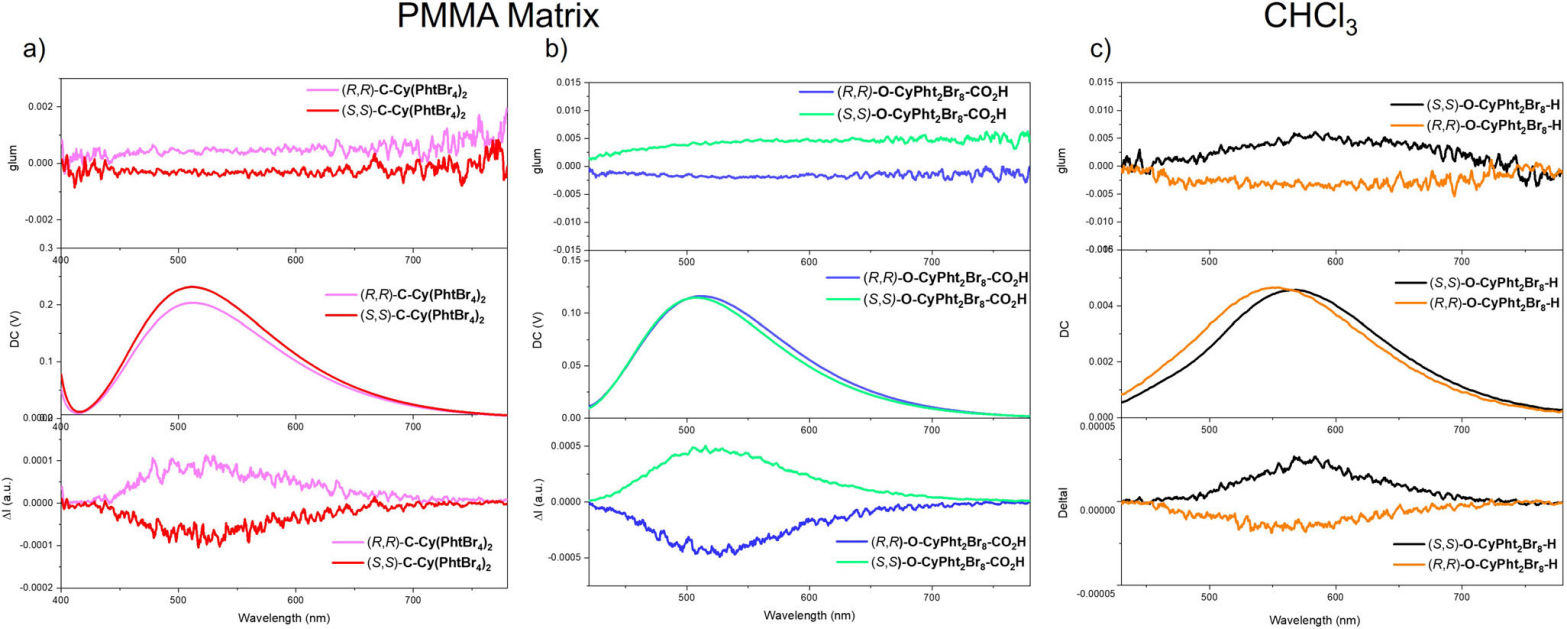
Luminescent dissymmetry factor *g*_lum_ (top) and CPL spectra (bottom) of 5% doped PMMA films of a) (*R*,*R*)‐**C‐Cy(PhtBr_4_)_2_** (pink) and (*S*,*S*)‐**C‐Cy(PhtBr_4_)_2_** (red) and b) (*R*,*R*)‐**O‐CyPht_2_Br_8_‐CO_2_H** (blue) and (*S*,*S*)‐**O‐CyPht_2_Br_8_‐CO_2_H** (green) under air at 298 K; c) Luminescent dissymmetry factor *g*_lum_ (top) and CPL spectra (bottom) of (*R*,*R*)‐**O‐CyPht_2_Br_8_‐H** (orange) and (*S*,*S*)‐**O‐CyPht_2_Br_8_‐H** (black) in chloroform at RT.

Our novel materials systems enable the unprecedented possibility to investigate and compare the CPL of chiral RTP both in solid state and in solution. We firstly investigated the CPL response of doped PMMA films of these compounds and compared the CPL signal of **C‐Cy(PhtBr_4_)_2_** and **O‐CyPht_2_Br_8_‐CO_2_H** emitters with the one arising from **O‐CyPht_2_Br_8_‐H** aggregates (Figures 4 and S18). As depicted in Figure 4a–c, reliable mirror‐image spectra were obtained for all the compounds with *g*_lum_ of ca. 0.5 × 10^−3^ for **C‐Cy(PhtBr_4_)_2_** and 4.0 × 10^−3^ for both **O‐CyPht_2_Br_8_‐H** and **O‐CyPht_2_Br_8_‐CO_2_H**. The obtained value for **C‐Cy(PhtBr_4_)_2_** is in line with the intensity usually measured for the CP fluorescence of the chiral diamine unit.^[^ ^48^, ^49^, ^50^ ^]^ In contrast, the sign change and the higher value obtained for the two other systems highlight a more interesting picture.

**Figure 5:**
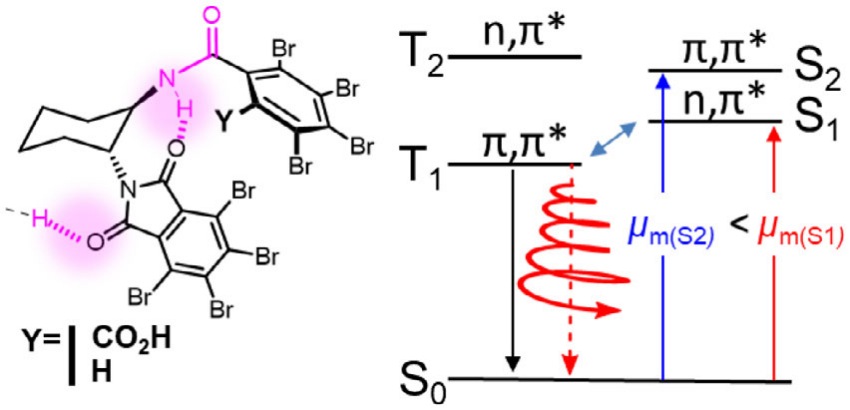
Simplified picture of the triplet–singlet excited states mixing and its impact on the intensity of CP phosphorescence (blue arrows represent the main SOC channel).

Following El‐Sayed's arguments,^[^ ^7^ ^]^ the phosphorescence emission is mainly driven by the coupling of the triplet excited‐state with the singlet one from which the SOC is significant. Based on these aspects, the phosphorescence for all three compounds may result primarily from a singlet n‐π* admixture. Regarding CP phosphorescence, different singlet/triplet interactions may impact the *g*_lum_ intensity due to the variations of the electric and magnetic dipole transition moments related to the admixed singlet transition. Indeed, knowing that singlet n‐π* transitions have often a more intense magnetic dipole than π‐π*, and vice versa for the electric dipole, a triplet emitting state that couples more strongly with a singlet n‐π* transition would acquire higher polarization than one coupling with a singlet π–π* transition. These theoretical arguments were put forward by Blok and Dekkers 35 years ago (Figure 5), and only explored for a limited number of chiral emitting phosphorescent systems.^[^ ^51^ ^]^ While it is rather difficult to rationalize the obtained CPL signal and the underlying process at play, this approximation is interesting to consider for tentatively explaining the rather higher *g*_lum_ observed for both **O‐CyPht_2_Br_8_‐CO_2_H** and **O‐CyPht_2_Br_8_‐H**. It is then surprising that the close system does not follow that trend despite a similar π‐π* character of its triplet state. However, the latter is delocalized on the two phthalimide unit, which clearly impacts its spin‐orbit coupling behavior (Figure 2) and the related electric and magnetic transition dipole moments for the phosphorescence transition, which may not be optimally oriented to reach high rotatory strengths. The presence of excitonic coupling can also induce mixing of the lowest emitting triplet state with other singlet ones, which may decrease the pure correspondence between phosphorescence *g*_lum_ and *g*_abs_ of the singlet transition.

Finally, CP phosphorescence was also recorded in CHCl_3_ solution for the three derivatives (Figure S17). However, only **O‐CyPht_2_Br_8_‐H** gave reliable CPL signals, with *g*_lum_ value up to 5.0 × 10^−3^ (Figure 4c). The similar intensity and sign obtained for the polarized emission in comparison with the one recorded for the PMMA film (Figure S18) further support the importance of the **O‐CyPht_2_Br_8_‐H** aggregates for obtaining CP‐RTP, which provides one of the rare examples of CP‐RTP in solution.^[^ ^33^, ^52^ ^]^

## Conclusion

In conclusion, we described the synthesis of original chiral RTP emitters based on phthalimides and investigated their polarized emission. First, we showed that the investigated compounds display efficient RTP in solid state, as well as in solution, affording emission with PLQY up to 15% and lifetimes going from the micro‐ to the millisecond timescales, due to the presence of the eight heavy bromine atoms. Beyond the effect of these atoms on the ISC, we revealed how the degree of freedom given to the structure plays a crucial role in the luminescent process by leveraging the creation of new intra/intermolecular interactions. These interactions not only impact the dynamic of the triplet excited state involved in the phosphorescence emission but also provide an efficient strategy to promote RTP in fluid media, resulting in unprecedented chiroptical properties. Especially, these interactions also modify the nature of the triplet excited‐state by means of n‐π* and π–π* characters and allow a different mixing with the singlet n‐π* and π–π* excited‐states, ultimately impacting the intensity of the CP‐RTP. While this picture remains a simplification of the underlying photophysical dynamics, it is interesting to note that this approach is verified on the chiral phthalimide systems developed in this study. These results may offer new opportunities to design innovative and efficient CP‐RTP while investigating their potential in bioimaging and CP‐OLED, among other applications. Ultimately, this study provides consistent elements to achieve a better understanding of the key parameters governing the polarized luminescence.

## Conflict of Interests

The authors declare no conflict of interest.

## Supporting information



Supporting Information

Supporting Information

## Data Availability

The data that support the findings of this study are available in the Supporting Information of this article.
