# Supplementary material for: Symmetry Breaking and Hydrogen Bonding in Phthalimide Compounds Enable Efficient Room‐Temperature Circularly Polarized Phosphorescence in Solution
Source: Angew Chem Int Ed Engl. 2025 Oct 9;64(48):e202515218. doi: 10.1002/anie.202515218 (PMC12643333; doi:10.1002/anie.202515218)
Supplement: Supplementary file 1 — Supporting Information [file ANIE-64-e202515218-s001.pdf]

Supporting Information

Symmetry Breaking and Hydrogen Bonding in Phthalimide compounds Enable Efficient Room-Temperature Circularly Polarized Phosphorescence in Solution

Catherine Demangeat,[a] Maxime Remond,[b] John M. Hudson,[c] Emrys W. Evans,[c] Denis Jacquemin,*[d,e] and Ludovic Favereau*[a]

[a] Dr. Catherine Demangeat, , Dr. Ludovic Favereau
Univ Rennes, CNRS, ISCR - UMR 6226,
F-35000 Rennes, France.
E-mail: ludovic.favereau@univ-rennes1.fr
[b] Dr. Maxime Remond, Univ Angers, CNRS, MOLTECH-Anjou, SFR MATRIX, F-49000 Angers, France
[c] Department of Chemistry, Swansea University, Swansea SA2 8PP, United Kingdom; Centre for Integrative Semiconductor Materials, Swansea SA1 8EN, United Kingdom
[d] Prof. Denis Jacquemin, Nantes Université, CNRS, CEISAM UMR 6230, F-44000 Nantes, France. E-mail : Denis.Jacquemin@univ-nantes.fr
[e] Institut Universitaire de France (IUF), F-75005, Paris, France

Table of Contents

A. General Methods **p2**
B. Synthesis **p3**
C. Photophysical characterizations **p6**
D. Chiroptical properties **p12**
E. FTIR **p14**
F. Single crystal X-Ray diffraction analysis **p15**
G. NMR data **p16**
H. Theoretical calculations **p24**
I. Transient absorption study **p38**

References

A. General Methods

Experimental methods

^{1}H and ^{13}C NMR spectra were recorded at room temperature on an *AVANCE III 300 BRUKER* or an *AVANCE III 400 BRUKER* at Centre Régional de Mesures Physiques de l'Ouest (CRMPO), Université de Rennes 1. Chemical shifts δ are given in ppm, relative to an internal standard of residual deuterated solvent ($CDCl_3$: δ = 7.26 ppm for ^{1}H NMR, δ = 77.16 ppm for ^{13}C NMR, DMSO-d_6: δ = 2.05 ppm for ^{1}H NMR, δ = 39.52 ppm for ^{13}C NMR); and coupling constants *J* in Hz.

High-resolution mass (HR-MS) determinations were performed at CRMPO on a Bruker MaXis 4G by ASAP (+ or -), ESI (with dichloromethane/methanol as solvents system) or MALDI techniques. Experimental and calculated masses are given with consideration of the mass of the electron.

UV-Visible (UV-vis, in M^{-1} cm^{-1}) absorption spectra were recorded on a V-730 JASCO spectrophotometer. Emission spectra were measured on an FP-8350 JASCO fluorimeter with a Xenon lamp. Absolute quantum yields were recorded using a deported HORIBA Scientific Quanta-Phi integrating sphere linked to the Fluoromax-4. The lifetime and time-resolved emission spectra were recorded on a HORIBA Scientific Fluoromax-4 equipped with its TCSPC pulsed source interface.

Electronic circular dichroism spectra (ECD, in M^{-1} cm^{-1}) were recorded on a Jasco J-1700 Circular Dichroism Spectrometer. Molar rotations are given in deg cm^2 $dmol^{-1}$.

The circularly polarized luminescence (CPL) measurements were performed using a JASCO 300 CPL spectrofluoropolarimeter. The following parameters were used: emission slit width ≈ 20 mm, integration time = 4 sec, scan speed = 50 nm/min, accumulations = 8. The concentration of all the samples was *ca*. 10^{-5}–10^{-6} M. Excitation of the samples was performed at 380 nm.

The crystals of **C-Cy(PhtBr$_4$)$_2$** were grown by slow evaporation of a biphasic mixture (Chloroform/Heptane). The structure was determined by standard crystallographic methods. These datas can be obtained on the Cambridge Crystallographic Data Centre via www.ccdc.cam.ac.uk/data_request/cif), the CCDC number is 2349404.

Reagents and materials

Unless otherwise stated, all reagents used in the experiments were purchased from commercial sources without further purification.

B. Synthesis

(*RR*)- and (*SS*)-**C-Cy(PhtBr$_4$)$_2$** were prepared in a straightforward synthesis following a similar procedure than the one we used in previous contributions.[1,2] The open forms (*R*,*R*)- or (*S*,*S*)-**O-CyPht$_2$Br$_8$-CO$_2$H** were obtained by the condensation reaction of the enantiopure (*RR*)- or (*SS*)-cyclohexane-1,2-diamine with tetrabromo phthalic anhydride in refluxing acetic acid. Further heating in DMF lead to the decarboxylation of the latter compounds and subsequently, the formation of (*R*,*R*)- or (*S*,*S*)-**O-CyPht$_2$Br$_8$-H.**

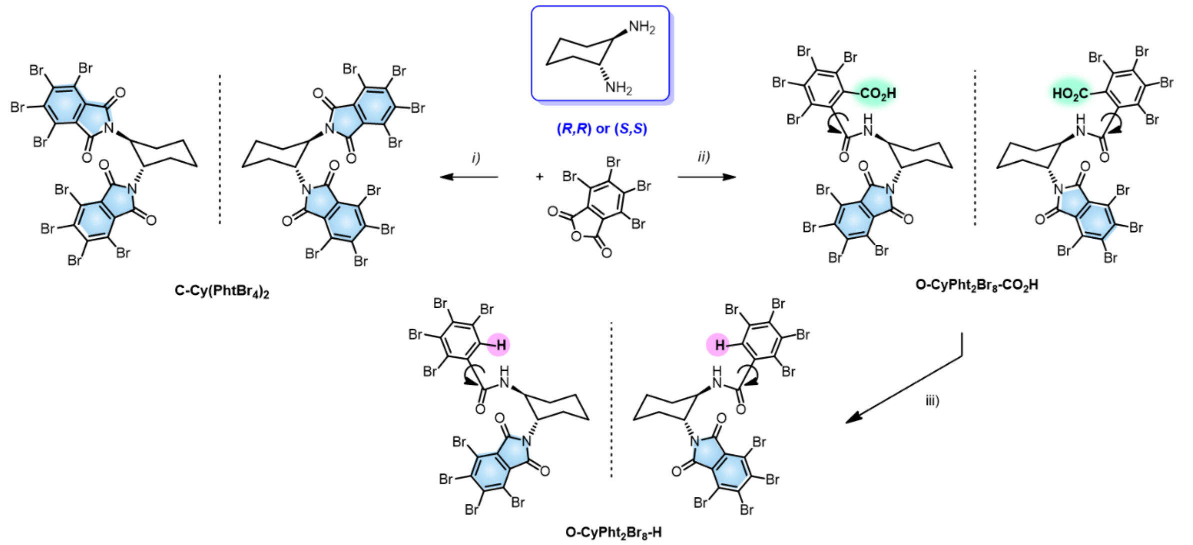

Scheme S1. Synthesis of (*R*,*R*)- or (*S*,*S*)-**C-Cy(PhtBr$_4$)$_2$,** (*R*,*R*)- or (*S*,*S*)-**O-CyPht$_2$Br$_8$-CO$_2$H** and (*R*,*R*)- or (*S*,*S*)-**O-CyPht$_2$Br$_8$-H** with the corresponding chemical structures; *i) toluene, Et$_3$N, reflux 5h; ii) refluxing acetic acid, 4h ; iii) DMF, reflux for 3.5h.*

Synthesis of (*R*,*R*)-2,2'-(cyclohexane-1,2-diyl)bis(4,5,6,7-tetrabromoisoindoline-1,3-dione) – (*R*,*R*)-C-Cy(PhtBr$_4$)$_2$

(*R*,*R*)-1,2-diaminocyclohexane (100 mg, 0.88 mmol) and 4,5,6,7-tetrabromoisobenzofuran-1,3-dione (850 mg, 1.84 mmol) were suspended in toluene (10 mL). NEt$_3$ (0.1 mL) was then added and the mixture was refluxed in a Dean-Stark for 5 hours. After cooling down, heptane was added and the precipitate was filtered on a frit and washed with pentane. Purification was done by column chromatography on silica gel with CHCl$_3$ as eluent to give the expected product as a yellow powder (670 mg, 0.67 mmol, 77 %). Spectra analyses (^{1}H and ^{13}C), and mass spectroscopy were similar to the results previously described.[2]

^{1}H NMR (400 MHz, CDCl$_3$): 5.07-4.92 (m, 2H), 2.49-2.33 (m, 2H), 1.98-1.84 (m, 4H), 1.54-1.45 (m, 2H).

^{13}C NMR (100 MHz, CDCl$_3$): 163.5, 121.5, 52.0, 29.1, 25.0.

(*S*,*S*)-**2,2'-(cyclohexane-1,2-diyl)bis(4,5,6,7-tetrabromoisoindoline-1,3-dione) –** (*S*,*S*)-**C-Cy(PhtBr$_4$)$_2$**

(*S*,*S*)-1,2-diaminocyclohexane (100 mg, 0.88 mmol) and 4,5,6,7-tetrabromoisobenzofuran-1,3-dione (850 mg, 1.84 mmol) were suspended in toluene (10 mL). NEt_3 (0.1 mL) was then added and the mixture was refluxed in a Dean-Stark for 5 hours. After cooling down, heptane was added and the precipitate was filtered on a frit and washed with pentane. Purification was done by column chromatography on silica gel with $CHCl_3$ as eluent to give the expected product as a yellow powder (440 mg, 0.44 mmol, 50 %). Spectra analyses (^{1}H and ^{13}C), and mass spectroscopy were similar to the results previously described.[2]

^{1}H NMR (400 MHz, $CDCl_3$): 4.95-4.80 (m, 2H), 2.18-2.00 (m, 2H), 1.98-1.76 (m, 4H), 1.46-1.30 (m, 2H).

^{13}C NMR (100 MHz, $CDCl_3$): 163.6, 121.6, 51.9, 29.1, 25.0.

HR-MS (ESI-QTOF) m/z: calcd for $C_{22}H_{11}N_2O_4Br_8$: 997.4108, found: 997.4118

Synthesis of 2,3,4,5-tetrabromo-6-(((1*S*,2*S*)-2**-(4,5,6,7-tetrabromo-1,3-dioxoisoindolin-2-yl)cyclohexyl)carbamoyl)benzoic acid –** (*S*,*S*)**-O-CyPht$_2$Br$_8$-CO$_2$H**

(*S*,*S*)-1,2-diaminocyclohexane (334 mg, 3.00 mmol) and 4,5,6,7-tetrabromoisobenzofuran-1,3-dione (2.85 mg, 6.15 mmol) were suspended in acetic acid (5 mL). The mixture was refluxed for 4 hours then cooled down to RT, and then to 4°C overnight. Water was added and the white precipitate was filtered and washed with water to give a white powder (267 mg, 2.61 mmol, 87 %).

^{1}H NMR (400 MHz, DMSO-d_6): 13.92 (s, broad, 1H), 8.58 (d, J = 8.0 Hz, 1H), 4.46 (m, 1H), 3.94 (dt, J_1 = 4.0 Hz, J_2 = 12.0 Hz, 1H), 2.16-1.97 (m, 2H), 1.88-1.70 (m, 3H), 1.41-1.25 (m, 3H).

^{13}C NMR (100 MHz, DMSO-d_6): 165.4, 163.8, 163.2, 138.1, 136.7, 130.9, 129.6, 129.3, 121.9, 121.4, 120.4, 54.2, 48.9, 31.5, 28.5, 24.6, 24.2.

HR-MS (ESI-QTOF) m/z: calcd for $C_{22}H_{12}N_2O_5Br_8Na^+$: 1038.4105, found: 1038.4107

Synthesis of 2,3,4,5-tetrabromo-6-(((1*R*,2*R*)**-2-(4,5,6,7-tetrabromo-1,3-dioxoisoindolin-2-yl)cyclohexyl)carbamoyl)benzoic acid –** (*R*,*R*)**-O-CyPht$_2$Br$_8$-CO$_2$H**

(*R*,*R*)-1,2-diaminocyclohexane (148 mg, 1.30 mmol) and 4,5,6,7-tetrabromoisobenzofuran-1,3-dione (1248 mg, 2.69 mmol) were suspended in acetic acid (6 mL). The mixture was refluxed for 2 hours then cooled down to RT, and then to 4°C overnight. Water was added and the white precipitate was filtered and washed with water to give a white powder (1152 mg, 1.13 mmol, 87 %).

^{1}H NMR (400 MHz, DMSO-d_6): 13.9 (s, broad, 1H), 8.58 (d, J = 8.2 Hz, 1H), 4.53-4.38 (m, 1H), 3.94 (dt, J_1 = 3.8 Hz, J_2 = 11.5 Hz, 1H), 2.19-1.94 (m, 2H), 1.89-1.69 (m, 3H), 1.46-1.20 (m, 3H)

^{13}C NMR (100 MHz, DMSO-d_6): 165.9, 164.3, 163.7, 138.6, 136.8, 131.4, 130.1, 129.9, 122.4, 121.9, 120.9, 54.6, 49.4, 32.0, 29.0, 25.1, 24.7.

HR-MS (ESI-QTOF) m/z: calcd for $C_{22}H_{12}N_2O_5Br_8Na^+$: 1038.4105, found: 1038.4105

Synthesis of 2,3,4,5-tetrabromo-N-((1*S*,2*S*)-2-(4,5,6,7-tetrabromo-1,3-dioxoisoindolin-2-yl)cyclohexyl)benzamide – (*S*,*S*)-O-CyPht$_2$Br$_8$-H

A suspension of (*S*,*S*)-**O-CyPht$_2$Br$_8$-CO$_2$H** (500 mg, 0.49 mmol) in dry DMF (40 mL) was heated under refluxed for 3.5 hours. After cooling down to room temperature, the yellow precipitate formed was filtered and washed with ethanol to give the expected product (*S*,*S*)-**O-CyPht$_2$Br$_8$-H** as a white powder (430 mg, 0.44 mmol, 93%).

^{1}H NMR (400 MHz, DMSO-d_6): 8.58 (d, J = 8.0 Hz, 1H), 7.41 (s, 1H), 4.41-4.29 (m, 1H), 3.93 (dt, J_1 = 4.0 Hz, J_2 = 12.0 Hz, 1H), 2.36-2.23 (m, 1H), 2.03-1.94 (m, 1H), 1.89-1.73 (m, 3H), 1.52-1.21 (m, 3H).

^{13}C NMR (100 MHz, DMSO-d_6): 164.7, 163.4, 140.7, 136.3, 130.5, 129.4, 128.4, 124.1, 121.3, 120.2, 54.9, 48.9, 31.1, 27.6, 24.5, 24.1.

HR-MS (ESI-QTOF) m/z: calcd for $C_{21}H_{12}N_2O_3Br_8Na$: 994.4207, found: 994.4202

Synthesis of 2,3,4,5-tetrabromo-N-((1*R*,2*R*)-2-(4,5,6,7-tetrabromo-1,3-dioxoisoindolin-2-yl)cyclohexyl)benzamide – (*R*,*R*)-O-CyPht$_2$Br$_8$-H

A suspension of (*R*,*R*)-**O-CyPht$_2$Br$_8$-CO$_2$H** (250 mg, 0.245 mmol) in dry DMF (20 mL) was heated under refluxed for 4 hours. After cooling down to room temperature, the yellow precipitate formed was filtered and washed with ethanol to give the expected product (*R*,*R*)-**O-CyPht$_2$Br$_8$-H** as a white powder (223 mg, 0.230 mmol, 93%).

^{1}H NMR (400 MHz, DMSO-d_6): 8.58 (d, J = 8.3 Hz, 1H), 7.41 (s, 1H), 4.43-4.35 (m, 1H), 3.99-3.88 (m, 1H), 2.33-2.28 (m, 1H), 2.03-1.98 (m, 1H), 1.88-1.76 (m, 3H), 1.49-1.24 (m, 3H).

^{13}C NMR (100 MHz, DMSO-d_6): 164.9, 163.6, 140.9, 136.5, 130.7, 129.6, 128.6, 124.3, 121.5, 120.4, 55.1, 49.1, 31.3, 27.8, 24.6, 24.2.

HR-MS (ESI-QTOF) m/z: calcd for $C_{21}H_{12}N_2O_3Br_8Na$: 994.4207, found: 994.4195

Synthesis of 4,5,6,7-tetrabromo-2-cyclohexylisoindoline-1,3-dione, CyPhtBr$_4$

Cyclohexylamine (100 mg, 1.0 mmol) and 4,5,6,7-tetrabromoisobenzofuran-1,3-dione (464 mg, 1.0 mmol) were suspended in acetic acid (5 mL). The mixture was refluxed for 4 hours then cooled down to RT. Water was added and the white precipitate was filtered and washed with water to give the expected product as a white powder (327 mg, 0.6 mmol, 60 %). Spectra analyses (^{1}H and ^{13}C), and mass spectroscopy were similar to the results previously described.[1]

^{1}H NMR (400 MHz, $CDCl_3$): 4.20-4.10 (m, 1H), 2.27-2.14 (m, 2H), 1.90 (d, J = 13.1 Hz, 2H), 1.74 (d, J = 12.7 Hz, 3H), 1.45-1.20 (m, 3H).

C. Photophysical properties

C.1-Solid state

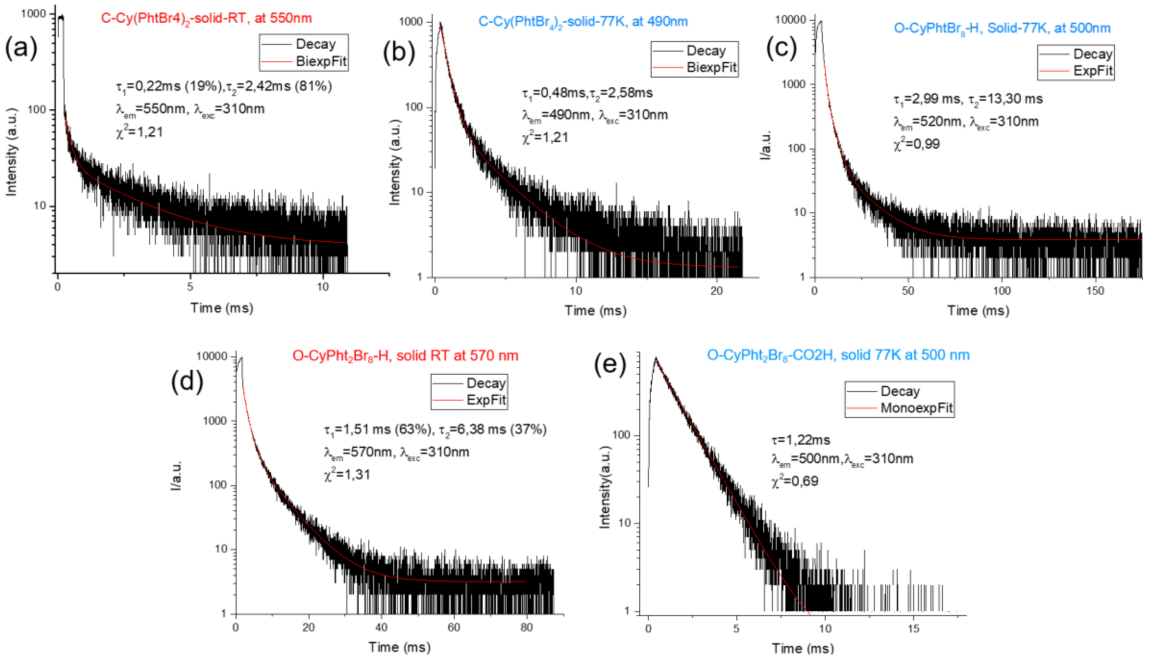

Figure S1. Lifetimes decay profiles of luminescence for the solids excited at 312 nm with the corresponding values of lifetimes (τ) for (a) **C-Cy(PhtBr$_4$)$_2$** at 298 K; (b) **C-Cy(PhtBr$_4$)$_2$** at 77 K and (c) **O-CyPht$_2$Br$_8$-H** at 77 K; (d) **O-CyPht$_2$Br$_8$-H** at 298 K and (e) **O-CyPht$_2$Br$_8$-CO$_2$H** at 77 K.

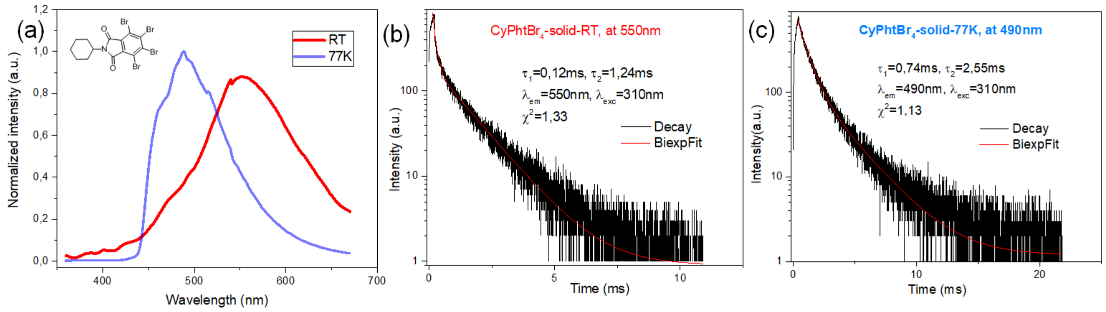

Figure S2. (a) PL spectra of **CyPhtBr$_4$** in the solid state at 298K (red line) and 77K (blue line) under 340 nm excitation; Lifetimes decay profiles with the corresponding values of lifetimes (τ) for **CyPhtBr$_4$** (b) at 298 K and (c) 77 K excited at 312 nm.

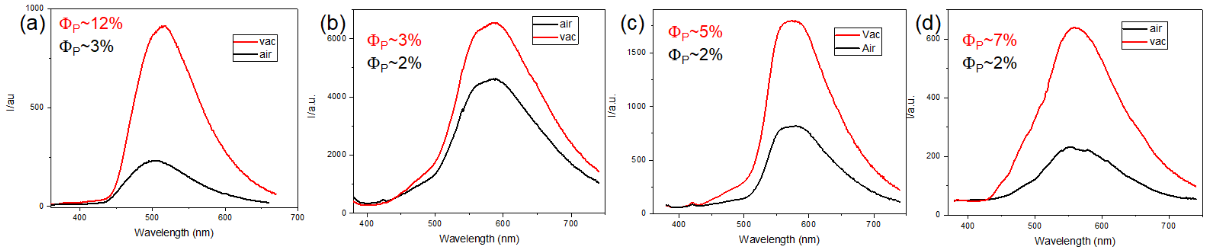

Figure S3. Effect of oxygen on the photoluminescence spectra (298 K) of (a) **O-CyPht$_2$Br$_8$-CO$_2$H** excited at 340 nm; (b) **C-Cy(PhtBr$_4$)$_2$;** (c) **O-CyPht$_2$Br$_8$-H**; and (d) **CyPhtBr$_4$** excited at 360 nm in the solid state at 298K (under air (black line) and vacuum conditions (red line)).

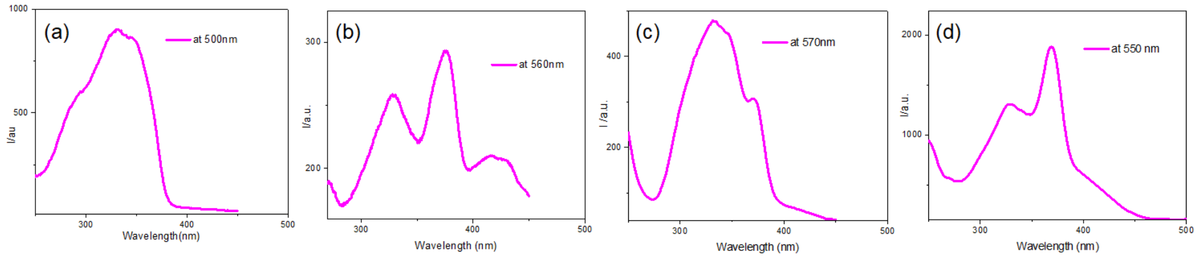

Figure S4. Excitation spectra in the solid state of (a) **O-CyPht$_2$Br$_8$-CO$_2$H** (at λ_{em}= 500 nm); (b) **C-Cy(PhtBr$_4$)$_2$** (at λ_{em}= 560 nm); (c) **O-CyPht$_2$Br$_8$-H** (at λ_{em}= 570 nm) and (d) **CyPhtBr$_4$** (at λ_{em}= 550 nm).

C.2-PMMA Films

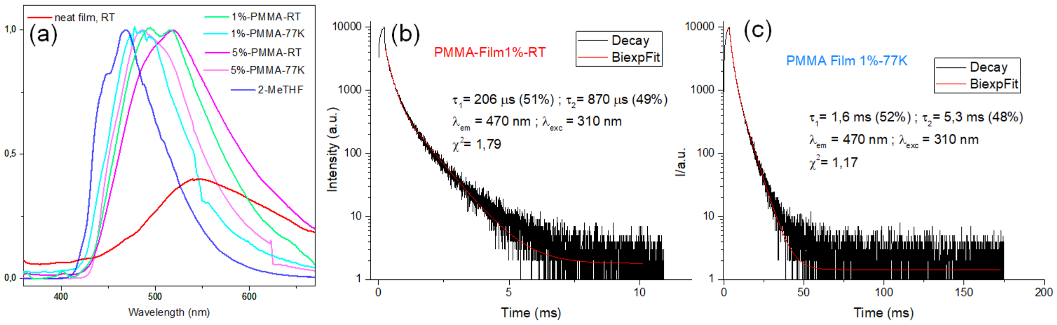

Figure S5. (a) Photoluminescence spectra of **CyPhtBr$_4$** dispersed in PMMA matrix with different doping rates and temperatures under 340 nm excitation; Lifetimes decay profiles with the corresponding values of lifetimes (τ) for **CyPhtBr$_4$** (b) at 298 K and (c) 77 K excited at 312 nm.

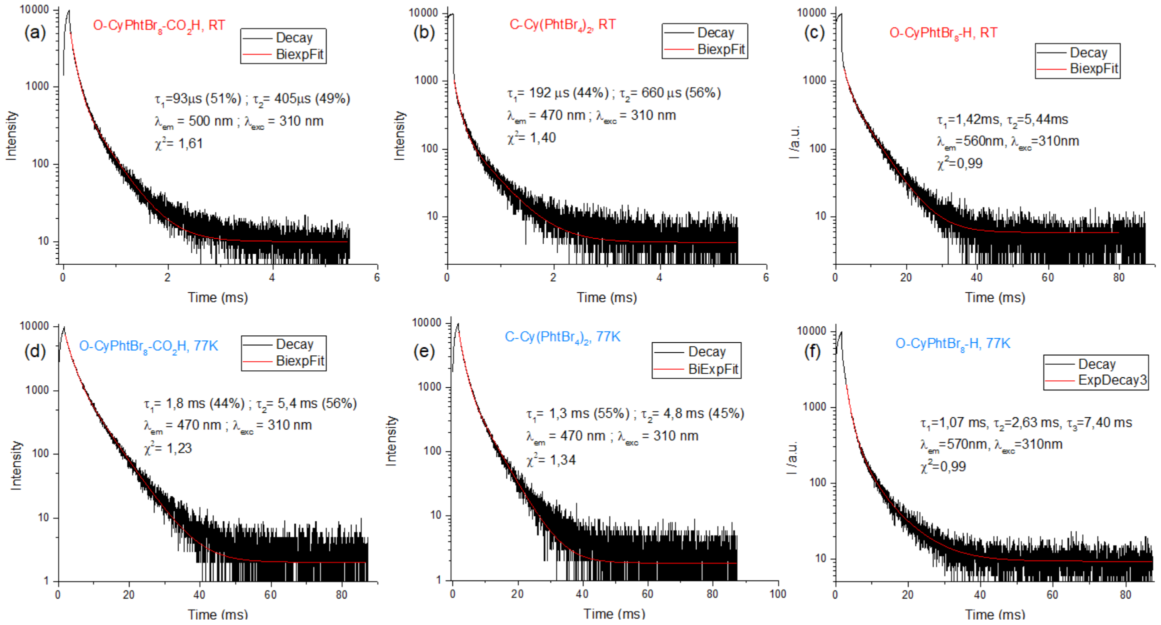

Figure S6. Lifetimes decay profiles of phosphorescence in PMMA matrix at 298 and 77 K with the corresponding values of lifetimes (τ) for (a,d) **O-CyPht$_2$Br$_8$-CO$_2$H**; (b,e) **C-Cy(PhtBr$_4$)$_2$** and (c,f) **O-CyPht$_2$Br$_8$-H** excited at 312 nm.

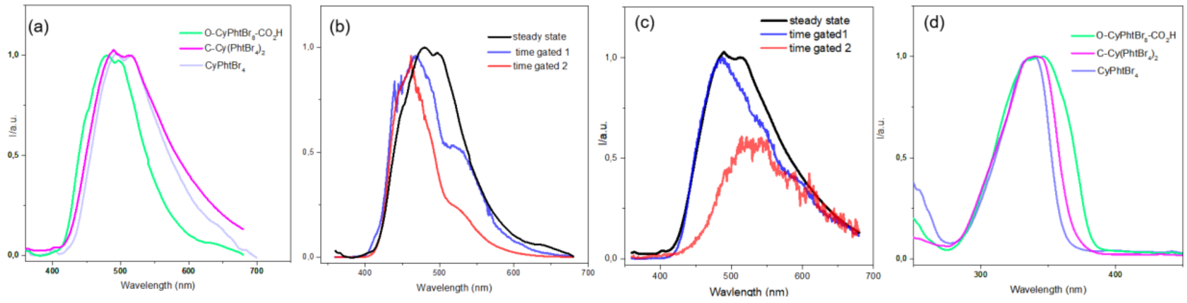

Figure S7. (a) Steady state PL spectra of **O-CyPht$_2$Br$_8$-CO$_2$H**, **C-Cy(PhtBr$_4$)$_2$** and **CyPhtBr$_4$** in PMMA matrix (1%wt) at 298K under 340 nm excitation; (b) Steady state and delayed emission spectra of **O-CyPht$_2$Br$_8$-CO$_2$H** (recorded with different time after excitation; time gated 1= integration time from 1 to 11 ms; time gated 2= integration time from 20 to 38 ms) in PMMA under 340 nm excitation at 77 K. (c) Steady state and delayed emission spectra of **C-Cy(PhtBr$_4$)$_2$** (recorded with different duration after excitation; time gated 1= integration time from 1 to 11 ms; time gated 2= integration time from 20 to 38 ms) in PMMA matrix (1%wt) under 340 nm excitation at 298 K. (d) Excitation spectra of **O-CyPht$_2$Br$_8$-CO$_2$H**, **C-Cy(PhtBr$_4$)$_2$** and **CyPhtBr$_4$** at λ_{em}=500 nm in PMMA matrix (1%wt) at 298K.

C.3-Solution

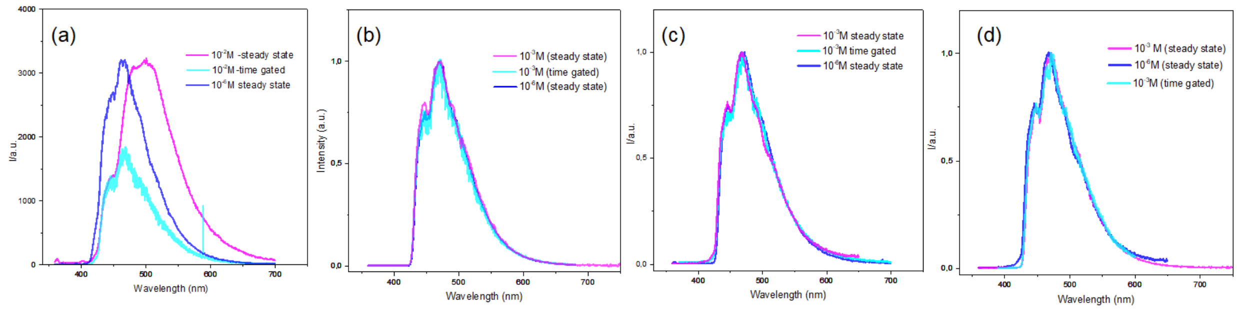

Figure S8. Steady state and time gated spectra (signal collected between 1 and 11 ms after excitation) recorded in 2-MeTHF glass at 77 K of (a) **O-CyPht$_2$Br$_8$-CO$_2$H** (b) **C-Cy(PhtBr$_4$)$_2$** (c) **O-CyPht$_2$Br$_8$-H** and (d) **CyPhtBr$_4$** at different concentrations (~10^{-6} M (blue line) and ~10^{-3} M (pink line)) excited at 340 nm.

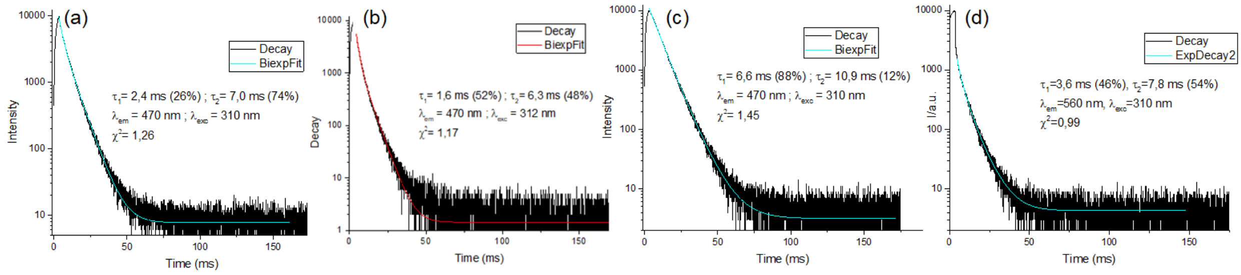

Figure S9. Lifetimes decay profiles of phosphorescence in frozen 2-MeTHF (77 K, 10^{-6}M) excited at 312 nm with the corresponding values of lifetimes (τ) for (a) **O-CyPhtBr$_8$-CO$_2$H**; (b) **C-Cy(PhtBr$_4$)$_2$;** (c) **O-CyPhtBr$_8$-H** and (d) **CyPhtBr$_4$**.

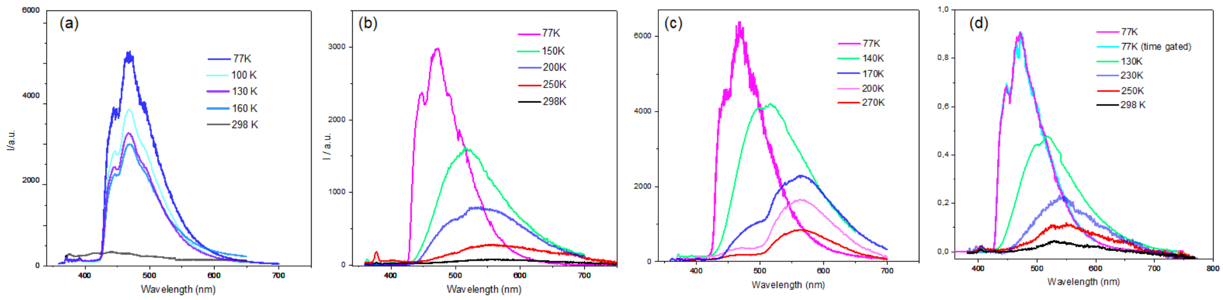

Figure S10. (a) Photoluminescence spectra of dilute 2-MeTHF (~10^{-6}M) solution recorded at different temperatures (from 77K to higher values); PL spectra in concentrated 2-MeTHF (~10^{-3}M) solution recorded at different temperatures (from 77K to higher values) of (b) **C-Cy(PhtBr$_4$)$_2$** (c) **O-CyPht$_2$Br$_8$-H** and (d) **CyPhtBr$_4$** excited at 340 nm.

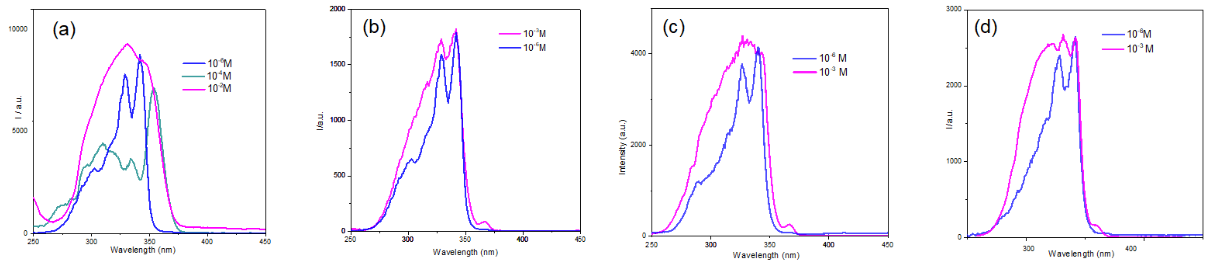

Figure S11. Excitation spectra of (a) **O-CyPht$_2$Br$_8$-CO$_2$H** (λ_{em}=500 nm); (b) **C-Cy(PhtBr$_4$)$_2$** (c) **O-CyPhtBr$_8$-H**; (d) **CyPhtBr$_4$** (λ_{em}=500 nm) in 2-MeTHF (77 K) at different concentrations (~10^{-6}M = blue line) and (~10^{-3}M = pink line).

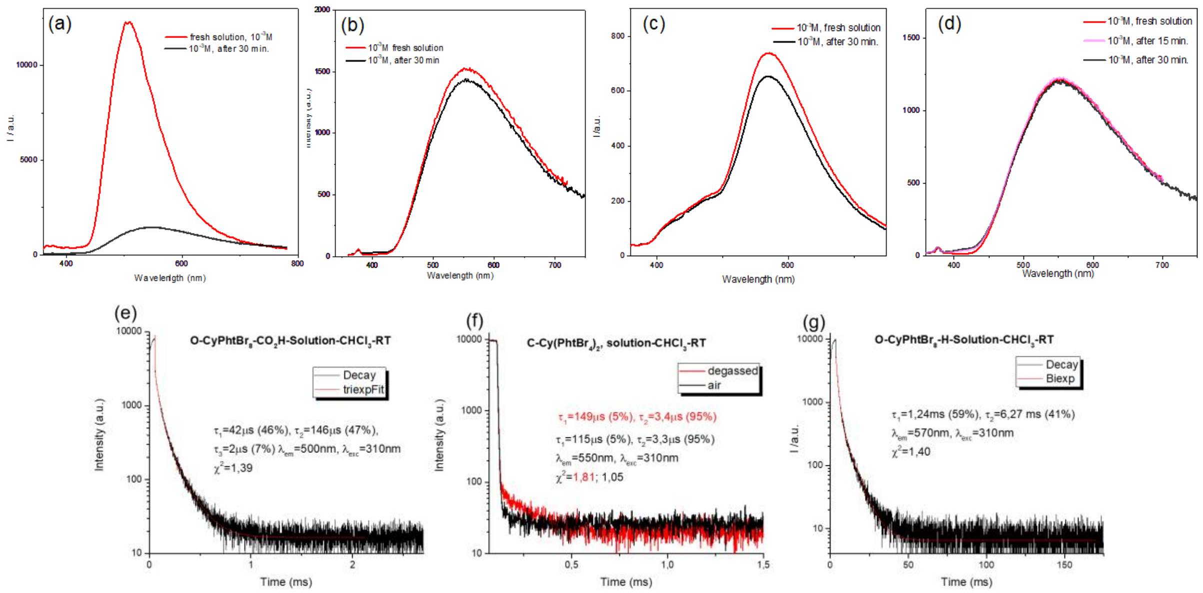

Figure S12. Photoluminescence spectra recorded in $CHCl_3$ solutions (10^{-3}M) at 298 K before (red line) and after 30 min. (black line) for (a) **O-CyPht$_2$Br$_8$-CO$_2$H**; (b) **C-Cy(PhtBr$_4$)$_2$** (c) **O-CyPht$_2$Br$_8$-H**; (d) **CyPhtBr$_4$** excited at 340 nm; Lifetimes decay profiles with the corresponding values of lifetimes (τ) for (e) **O-CyPht$_2$Br$_8$-CO$_2$H**; (f) **C-Cy(PhtBr$_4$)$_2$** ; (g) **O-CyPht$_2$Br$_8$-H**; (in aerated $CHCl_3$ solutions (10^{-3}M) at 298 K..

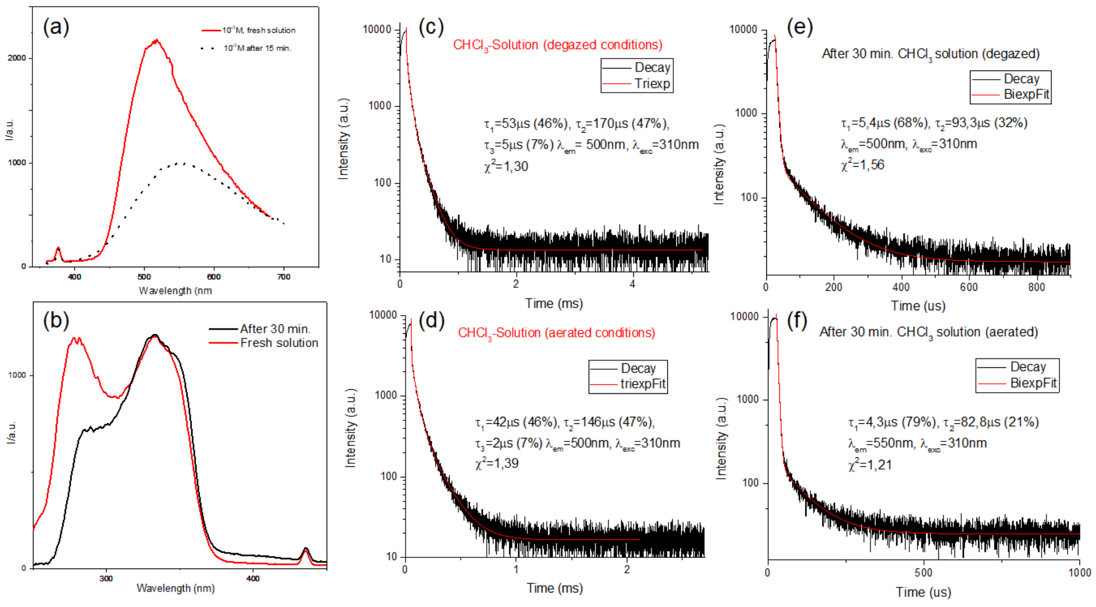

Figure S13. (a) Photoluminescence spectra of **O-CyPht$_2$Br$_8$-CO$_2$H** (298 K) in $CHCl_3$ solutions (10^{-3}M) and evolution of the signal after 15 min. (dashed black line) excited at 360 nm) ; (b) Excitation spectra **O-CyPht$_2$Br$_8$-CO$_2$H** (298 K) in $CHCl_3$ solutions (10^{-3}M) before (red line) and after 30 min. (black line) (λ_{em}=500 nm); Lifetimes decay profiles with the corresponding values of lifetimes (τ) for **O-CyPht$_2$Br$_8$-CO$_2$H** (298 K) in degassed $CHCl_3$ solutions (10^{-3}M) before (c) and after 30 min. (e); Lifetimes decay profiles with the corresponding values of lifetimes (τ) for **O-CyPht$_2$Br$_8$-CO$_2$H** (298 K) in aerated $CHCl_3$ solutions (10^{-3}M) before (d) and after 30 min. (f).

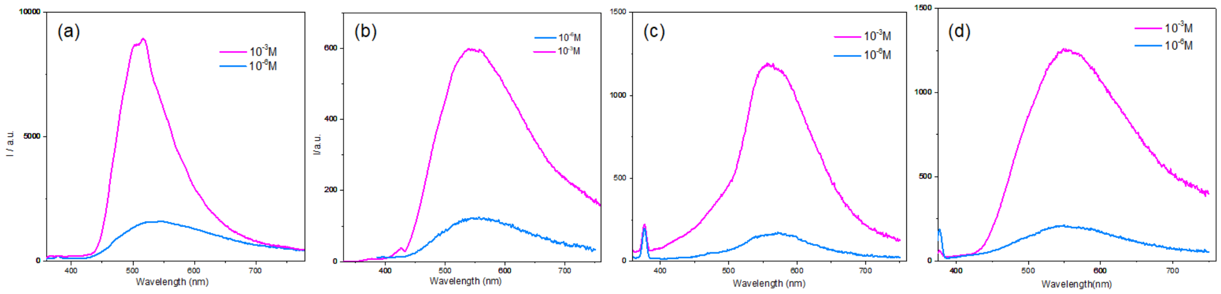

Figure S14. Photoluminescence spectra recorded in $CHCl_3$ solutions (298 K) at high (pink line) and low concentration (blue line) of (a) **O-CyPht$_2$Br$_8$-CO$_2$H**; (b) **C-Cy(PhtBr$_4$)$_2$** (c) **O-CyPht$_2$Br$_8$-H**; (d) **CyPhtBr$_4$** excited at 340 nm.

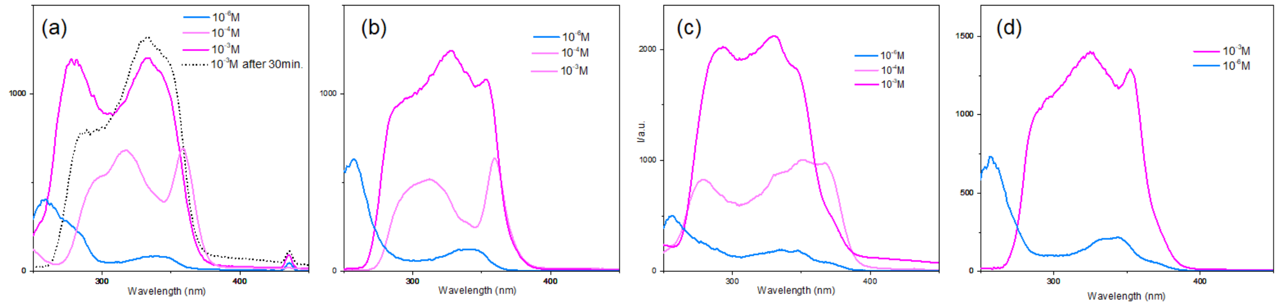

Figure S15. Excitation spectra of (a) **O-CyPht$_2$Br$_8$-CO$_2$H** (λ_{em}=500 nm); (b) **C-Cy(PhtBr$_4$)$_2$** (c) **O-CyPht$_2$Br$_8$-H**; (d) **CyPhtBr$_4$** (λ_{em}=550 nm) in $CHCl_3$ solutions (298 K) at different concentrations.

D. Chiroptical properties

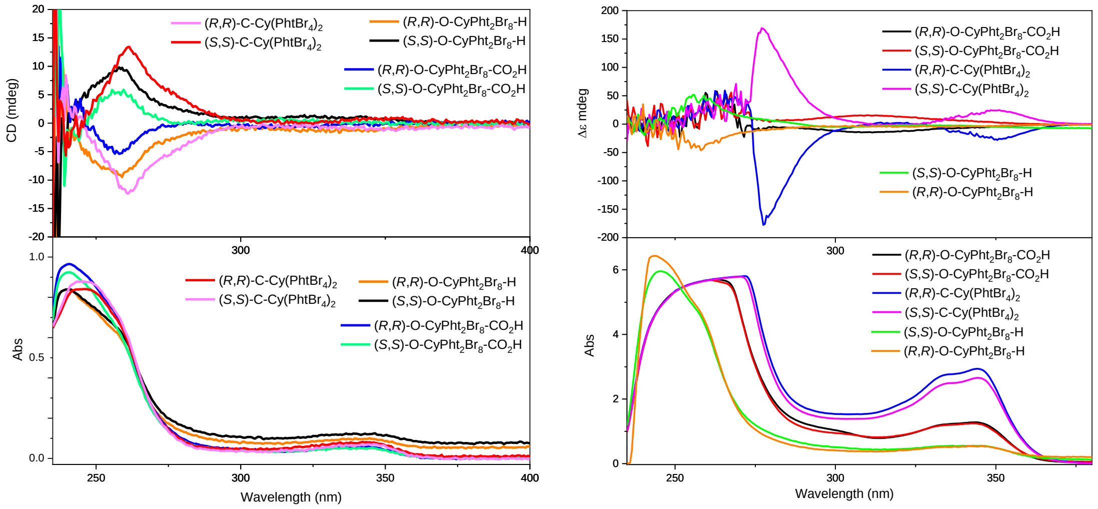

Figure S16. ECD (top) and Absorption (bottom) spectra of (*S*,*S*)- and (*R*,*R*)-**C-(CyPhtBr$_4$)$_2$**, **O-CyPht$_2$Br$_8$-CO$_2$H** and **O-CyPht$_2$Br$_8$-H** recorded on a Jasco J-1700 Circular Dichroism Spectrometer in $CHCl_3$ at ~10^{-6} M (left) and at ~10^{-2} M (right) at 298 K.

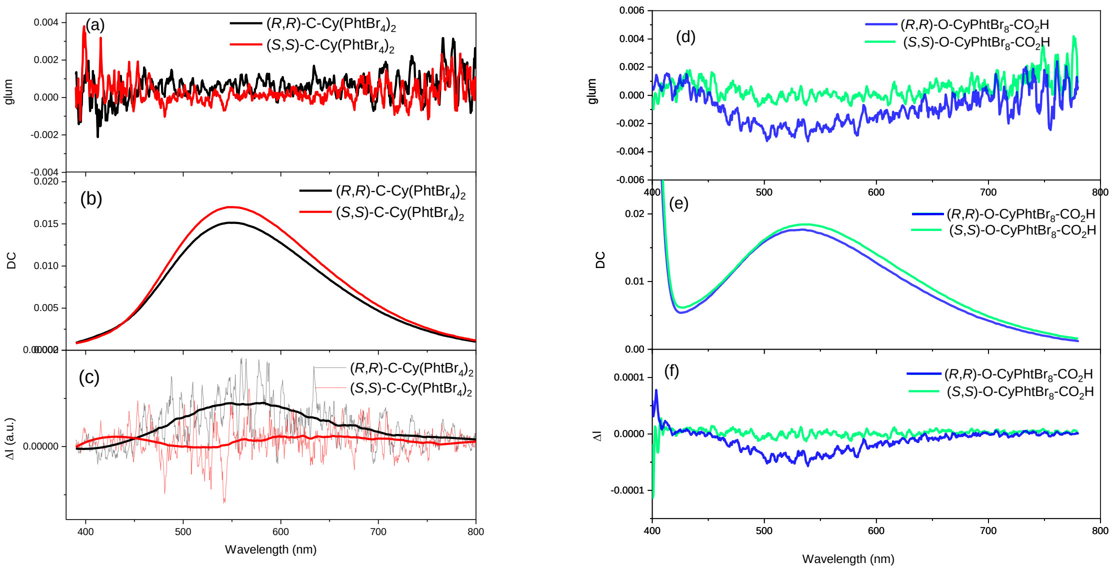

Figure S17. (a) Luminescent dissymmetry factor g_{lum} spectra of (*S*,*S*)-**C-Cy(PhtBr$_4$)$_2$** (red) and (*R*,*R*)-**C-Cy(PhtBr$_4$)$_2$** (black) in chloroform at RT. (b) and (c) CPL spectra of (*S*,*S*)-**C-Cy(PhtBr$_4$)$_2$** (red) and (*R*,*R*)-**C-Cy(PhtBr$_4$)$_2$** (black) in chloroform (10^{-3}M) under 360 nm excitation at 298 K. (d) Luminescent dissymmetry factor g_{lum} spectra of (*S*,*S*)-**O-CyPht$_2$Br$_8$-CO$_2$H** (green) and (*R*,*R*)-**O-CyPht$_2$Br$_8$-CO$_2$H** (blue) in chloroform at RT. (b) and (c) CPL spectra of of (*S*,*S*)-**O-CyPht$_2$Br$_8$-CO$_2$H** (green) and (*R*,*R*)-**O-CyPht$_2$Br$_8$-CO$_2$H** (blue) in chloroform (10^{-3}M) under 340 nm excitation at 298 K.

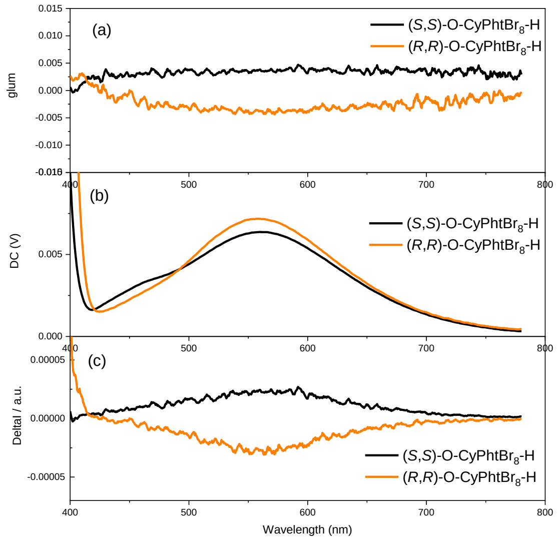

Figure S18. (a) Luminescent dissymmetry factor g_{lum} spectra of (*S*,*S*)-**O-CyPht$_2$Br$_8$-H** (black) and (*R*,*R*)-**O-CyPht$_2$Br$_8$-H** (orange) in PMMA matrix at RT under air. (b) and (c) CPL spectra of (*S*,*S*)-**O-CyPht$_2$Br$_8$-H** (black) and (*R*,*R*)-**O-CyPht$_2$Br$_8$-H** (orange) in PMMA matrix under 360 nm excitation at 298 K under air.

E. FTIR analysis.

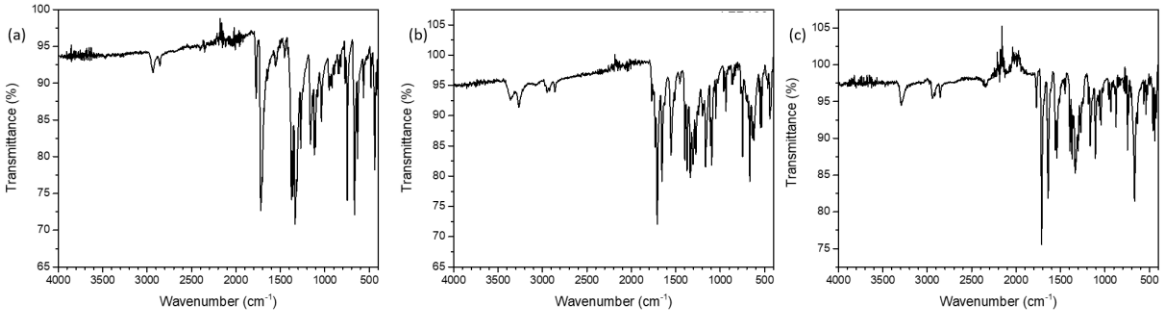

Figure S19. FT-IR spectra of **C-(CyPhtBr$_4$)$_2$** (a), **O-CyPht$_2$Br$_8$-CO$_2$H** (b), and **O-CyPht$_2$Br$_8$-H** (c) powders in KBr pellets.

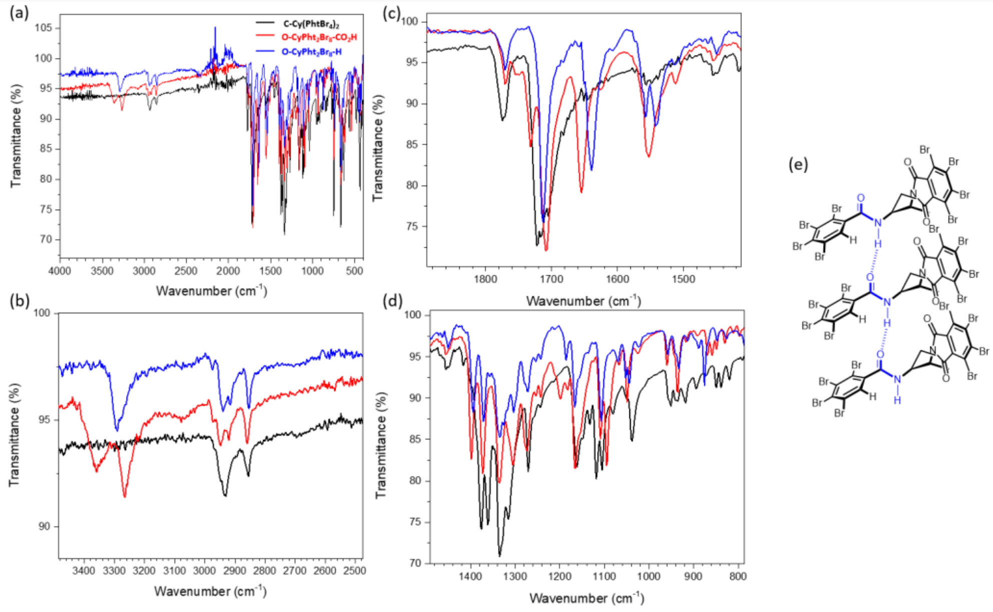

Figure S20. (a) FT-IR spectra of **C-(CyPhtBr$_4$)$_2$** (black line), **O-CyPht$_2$Br$_8$-CO$_2$H** (red line), and **O-CyPht$_2$Br$_8$-H** (blue line) powders in KBr pellets; (b) zoomed portion of the 3500-2500 cm^{-1} region; (c) zoomed portion of the 1900-1400 cm^{-1} region; (d) zoomed portion of the 1500-800 cm^{-1} region; (e) possible mode of self-assembly through H-bonding.

F. Single crystals X-Ray diffraction analysis.

Table S1. Crystal data and structure refinement for **C-Cy(PhtBr$_4$)$_2$**.

CCDC number	2349404	
Empirical formula	C22 H10 Br8 N2 O4	
Formula weight	1005.60 g/mol	
Temperature	150 (2) K	
Wavelength	0.71073 Å	
Crystal system	Monoclinic	
Space group	P21	
Unit cell dimensions	a = 9.1760(6) Å	
	b = 27.932(2) Å	β= 109.702(2)°.
	c = 13.4901(9) Å	
Volume	3255.2(4) Å^3	
Z	4	
Density (calculated)	2.052 g/cm^3	
Absorption coefficient	9.883 mm^{-1}	
F(000)	1872	
Crystal size	0.370 x 0.260 x 0.210 mm^3	
Crystal description	colourless prism	
Theta range for data collection	2.358 to 27.502°.	
Index ranges	-11<=h<=10, -36<=k<=36, -17<=l<=17	
Reflections collected/unique	26139/13092 [R(int) = 0.0468]	
Reflections [I>2sigma(I)]	11388	
Completeness to theta = 25.242°	0.987	
Absorption correction	multi-scan	
Max. and min. transmission	0.126 and 0.064	
Refinement method	Full-matrix least-squares on F^2	
Data / restraints / parameters	13092 / 1 / 607	
Goodness-of-fit on F^2	1.001	
Final R indices [I>2sigma(I)]	R1 = 0.0354, wR2 = 0.0719	
R indices (all data)	R1 = 0.0453, wR2 = 0.0749	
Extinction coefficient	n/a	
Largest diff. peak and hole	1.416 and -1.161 e.Å^{-3}	

G. NMR data

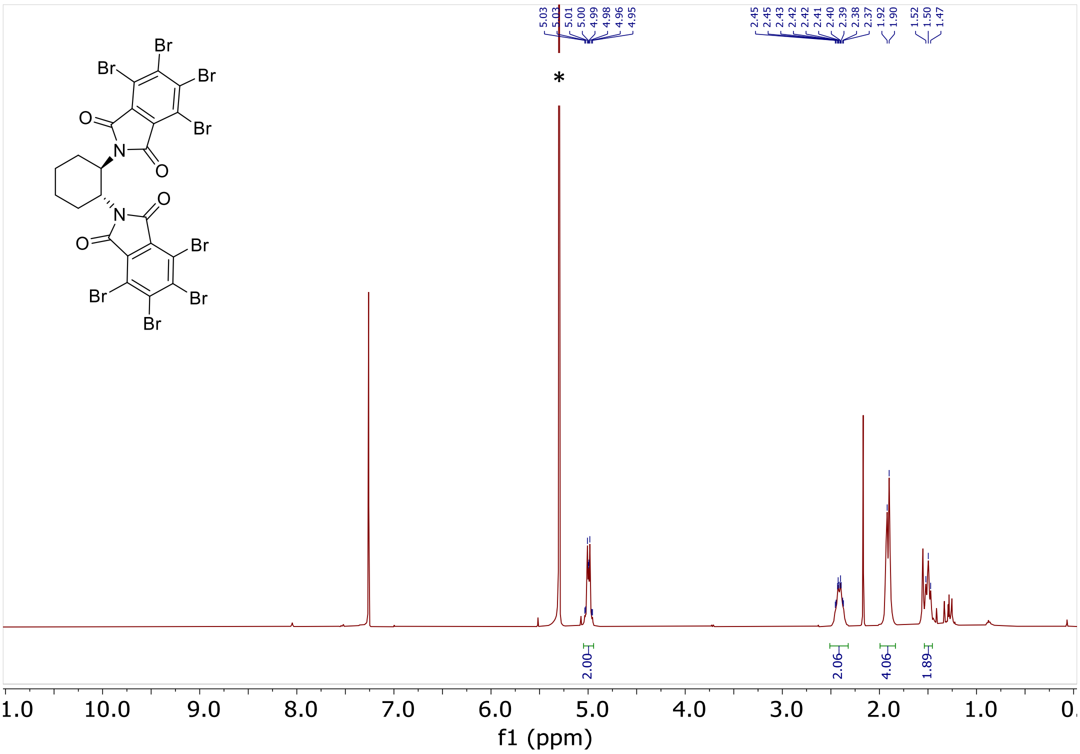

^{1}H NMR spectrum of compound *(R,R)*- **C-Cy(PhtBr4)2** in $CDCl_3$ at room temperature (400 MHz, * for residual CH_2Cl_2 signal).

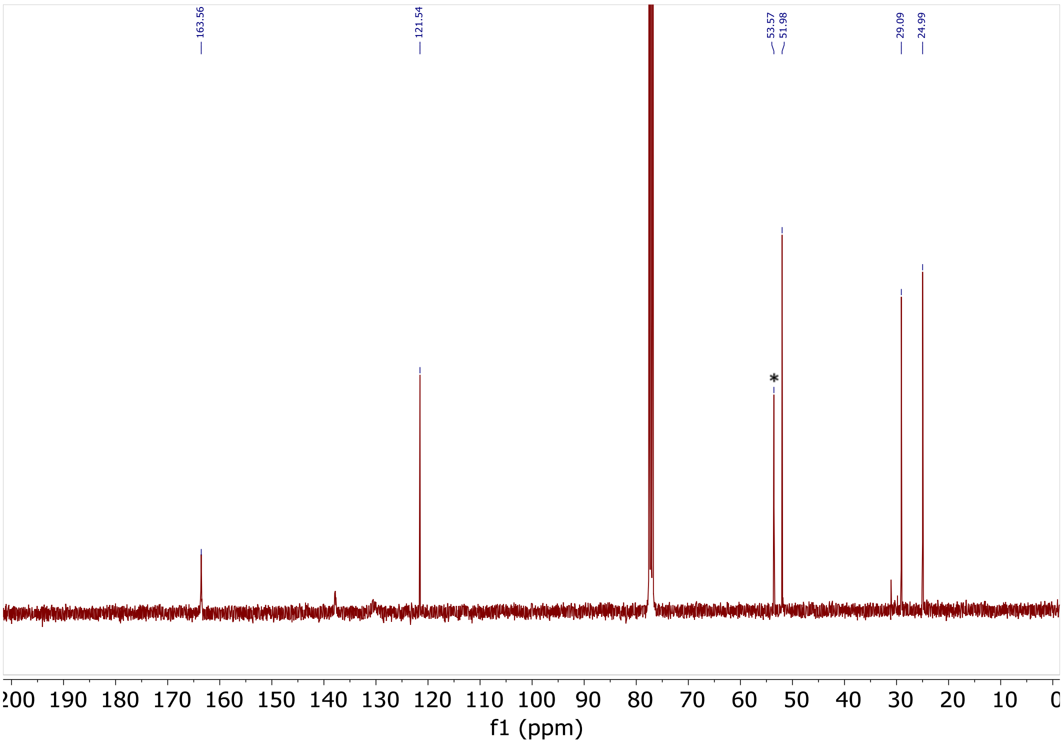

^{13}C NMR spectrum of compound *R,R*-**C-Cy(PhtBr4)2** in $CDCl_3$ at room temperature (100 MHz, * for residual CH_2Cl_2 signal).

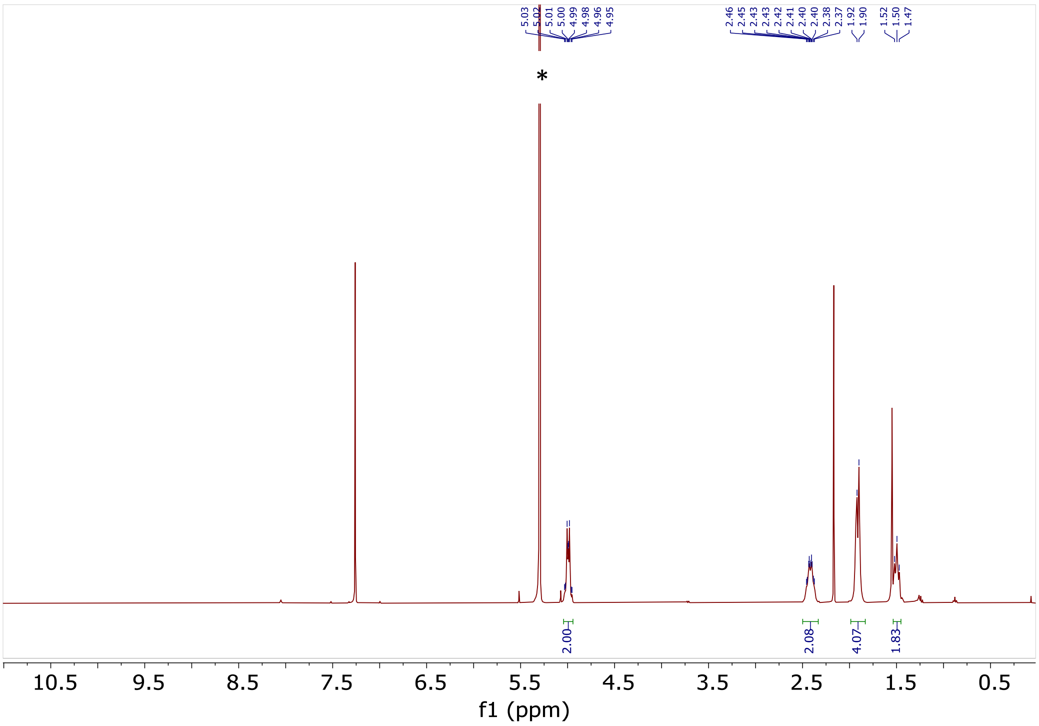

^{1}H NMR spectrum of compound (*S*,*S*)-**C-Cy(PhtBr$_4$)$_2$** in $CDCl_3$ at room temperature (400 MHz, * for residual CH_2Cl_2 signal).

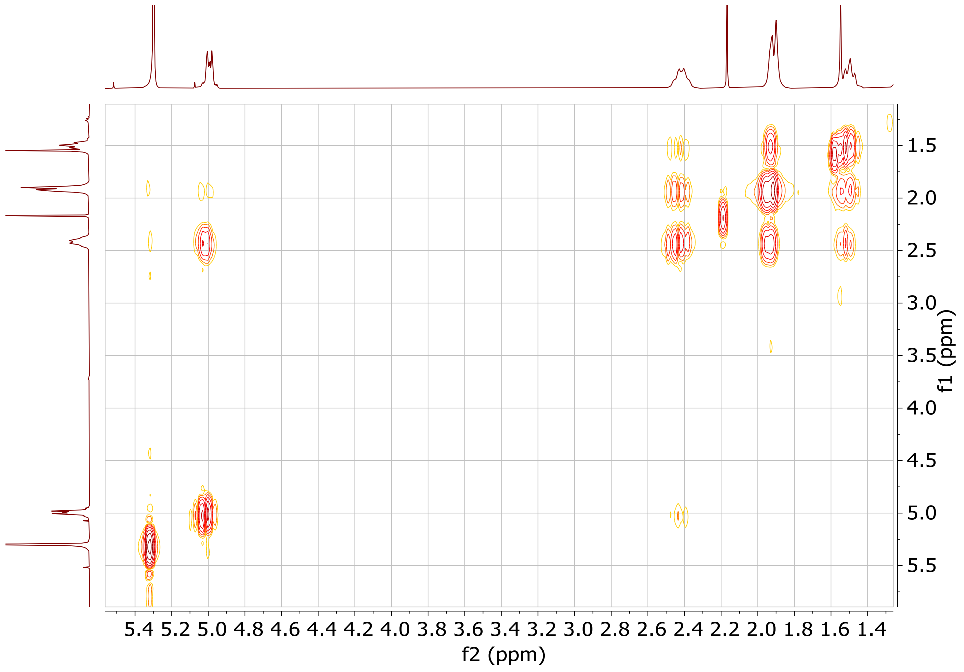

Region of the COESY spectrum of (*S*,*S*)-**C-Cy(PhtBr$_4$)$_2$** in a mixture of $CDCl_3$ and CD_2Cl_2 at room temperature (400 MHz).

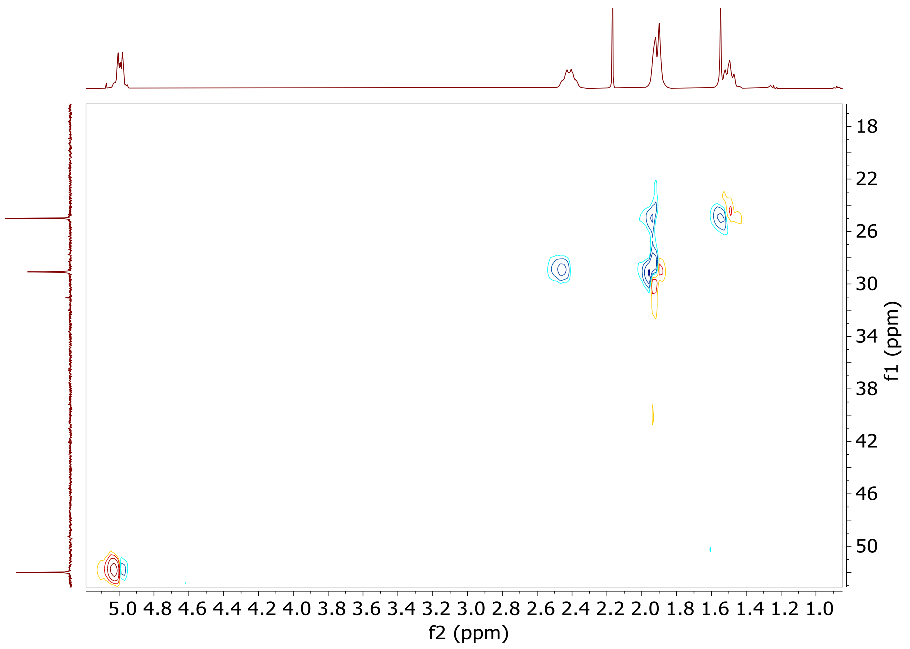

Region of the HSQC spectrum of (*S*,*S*)-**C-Cy(PhtBr$_4$)$_2$** in $CDCl_3$ at room temperature (400 MHz).

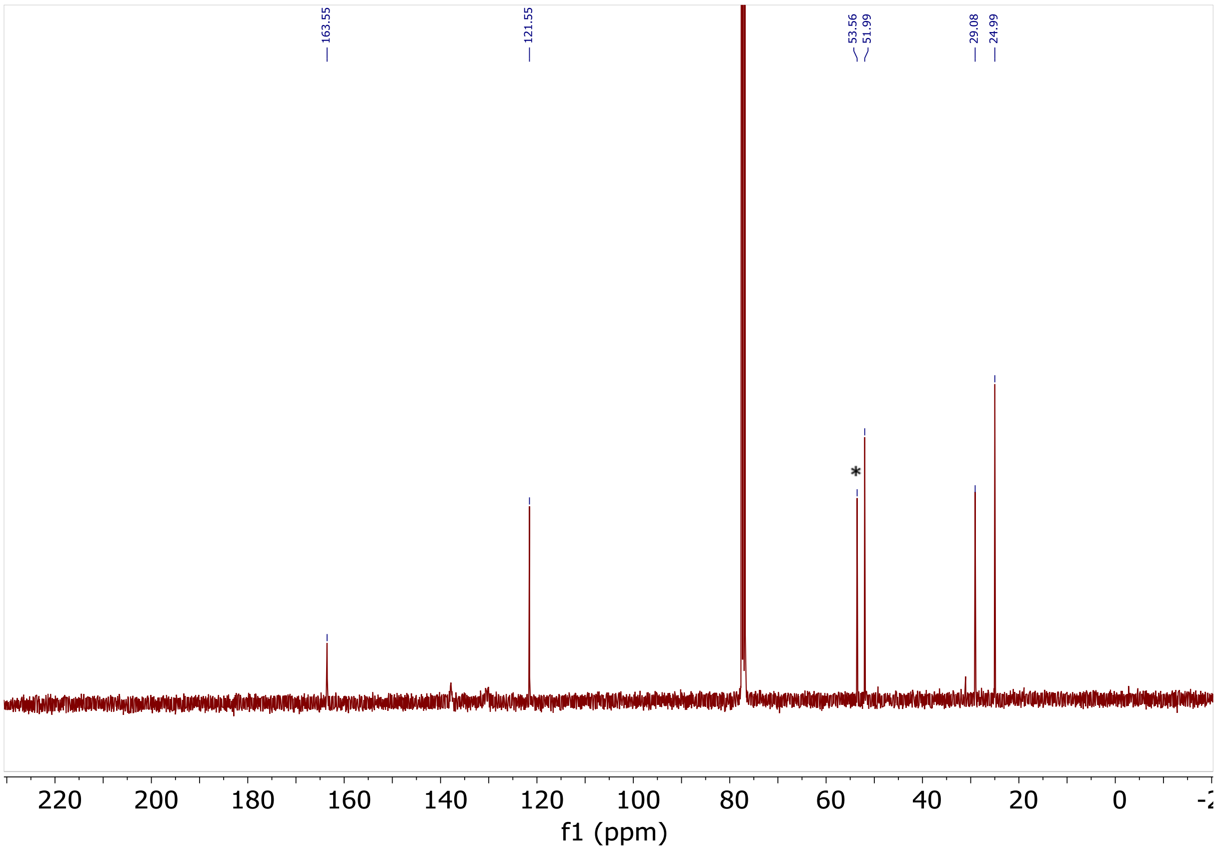

^{13}C NMR spectrum of compound (*S*,*S*)-**C-Cy(PhtBr$_4$)$_2$** in $CDCl_3$ at room temperature (100 MHz, * for residual CH_2Cl_2 signal).

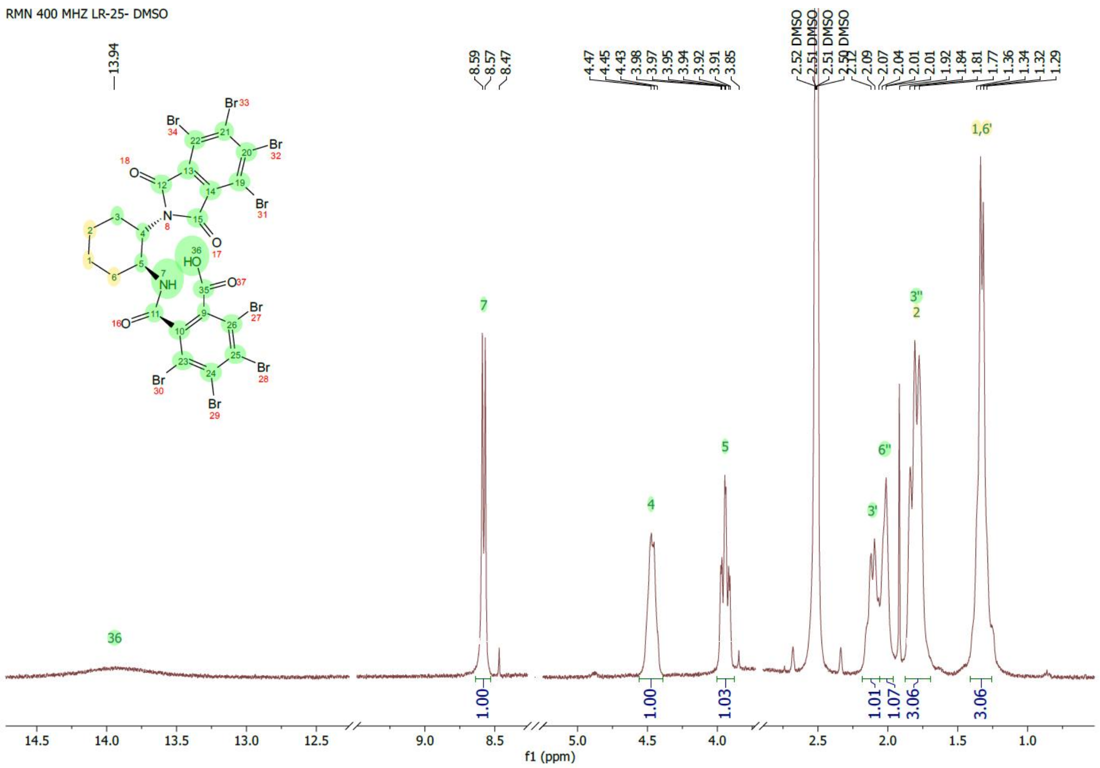

^{1}H NMR spectrum of compound *(R,R)*-**O-CyPht$_2$Br$_8$-CO$_2$H** in DMSO-d_6 at room temperature (400 MHz).

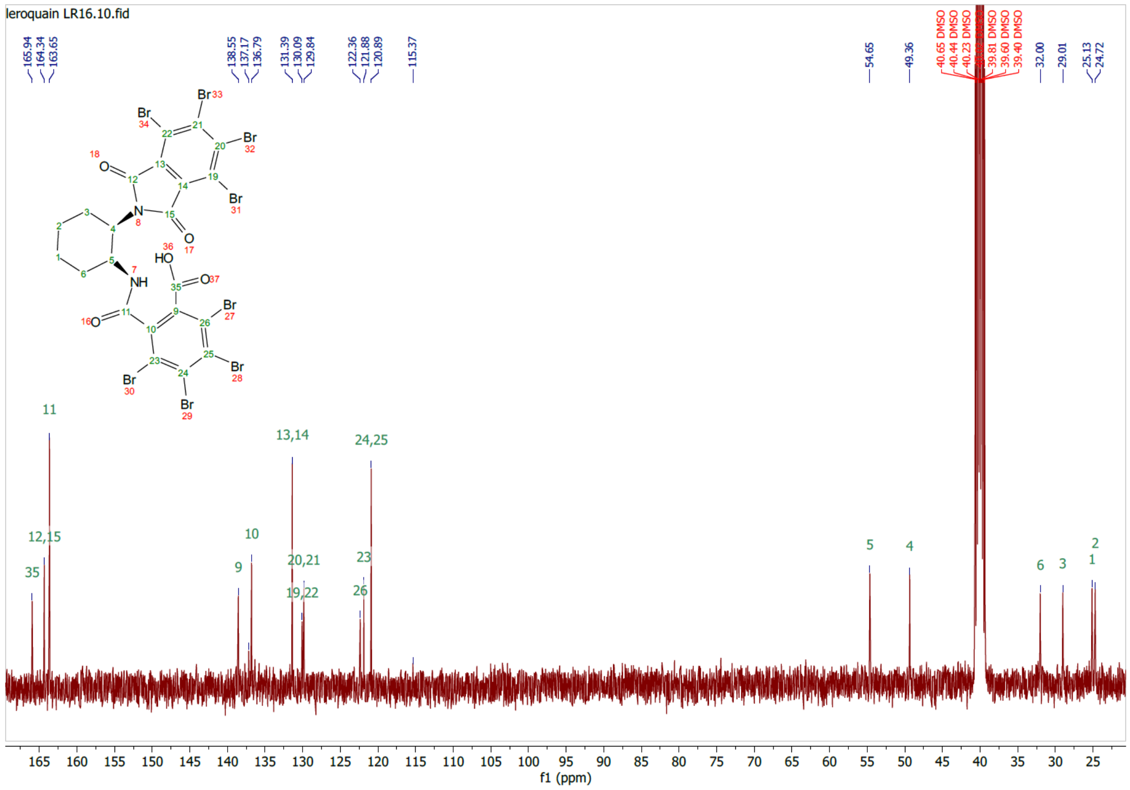

^{13}C NMR spectrum of compound *R,R* **O-CyPht$_2$Br$_8$-CO$_2$H** in DMSO-d_6 at room temperature (100 MHz).

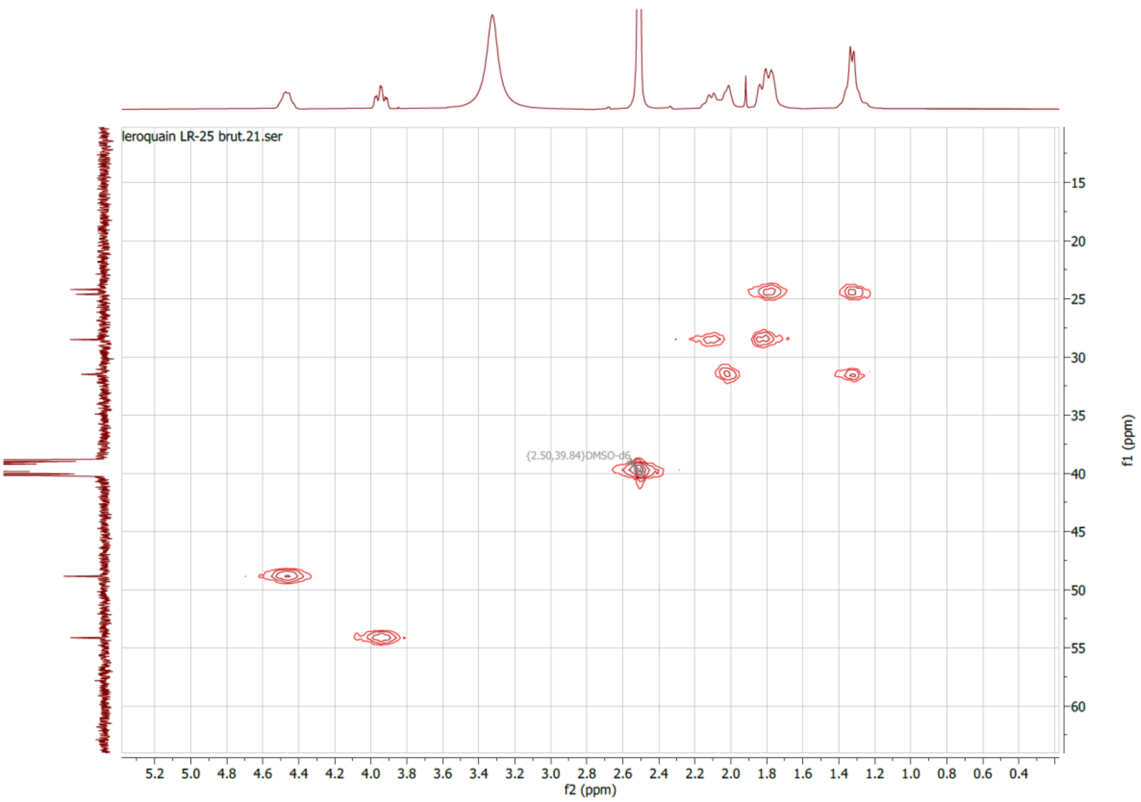

Region of the HMQC spectrum of *R*,*R* **O-CyPht$_2$Br$_8$- CO$_2$H** in DMSO-d_6 at room temperature.

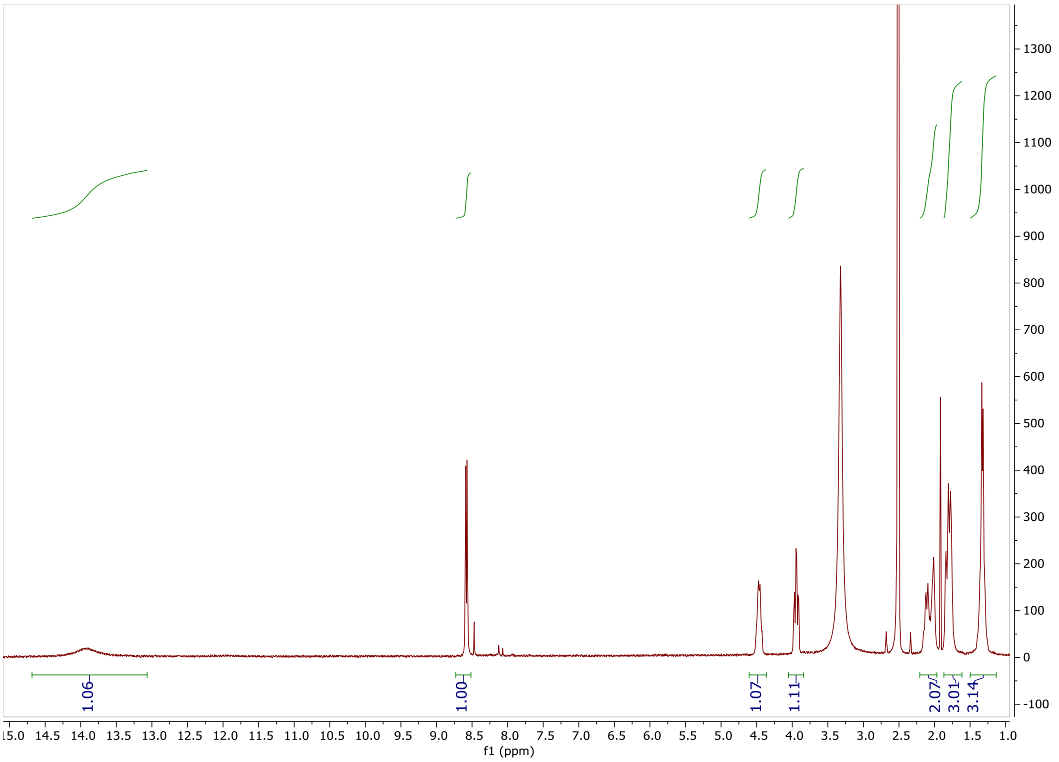

^{1}H NMR spectrum of compound (*S*,*S*)-**O-CyPht$_2$Br$_8$-CO$_2$H** in DMSO-d_6 at room temperature (400 MHz).

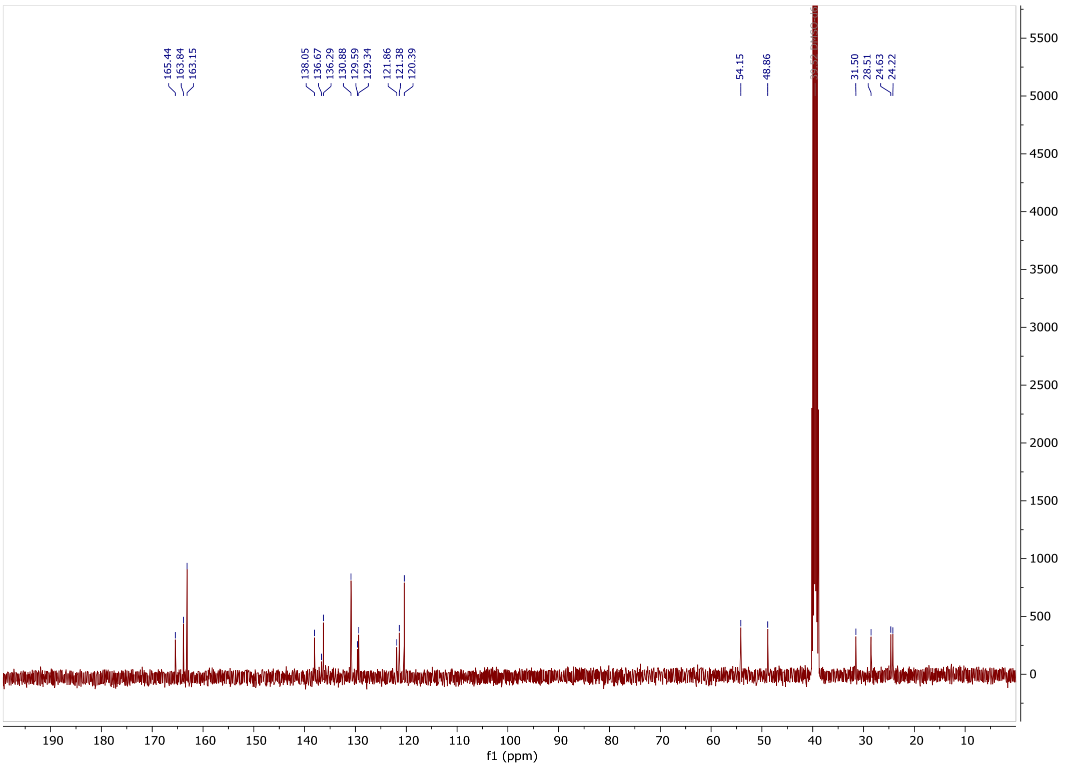

^{13}C NMR spectrum of compound *S,S* **O-CyPht$_2$Br$_8$-CO$_2$H** in DMSO-d_6 at room temperature (100 MHz).

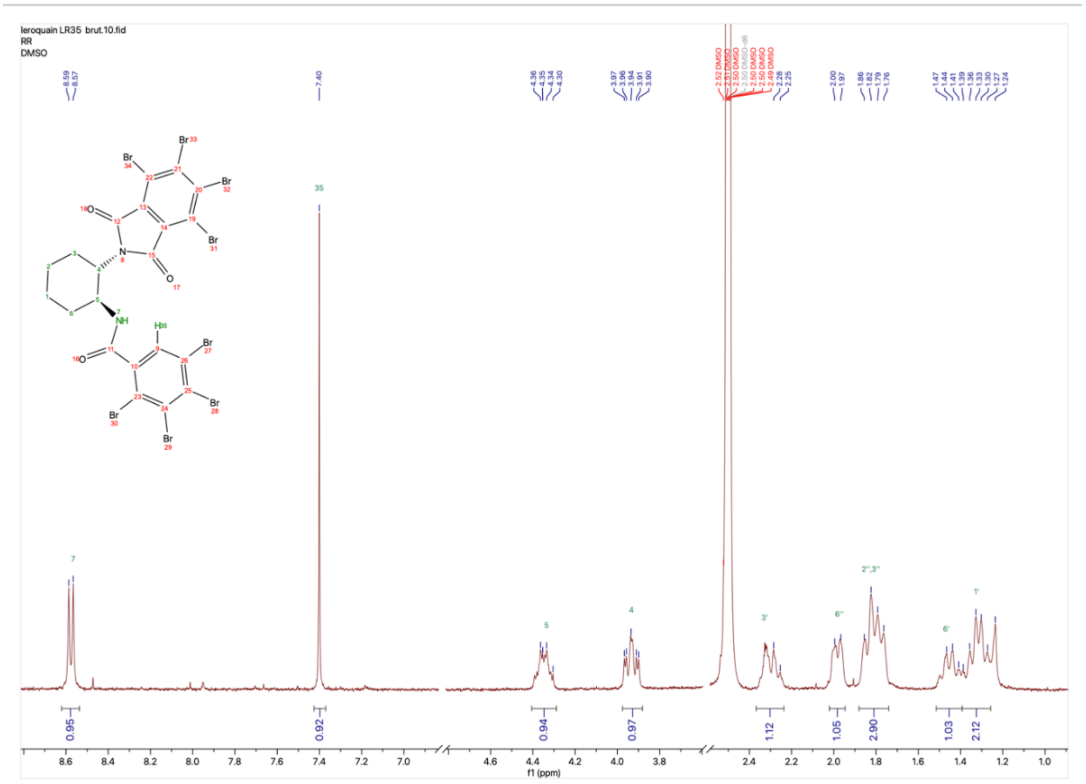

^{1}H NMR spectrum of compound *R,R* **O-CyPht$_2$Br$_8$-H** in DMSO-d_6 at room temperature (400 MHz).

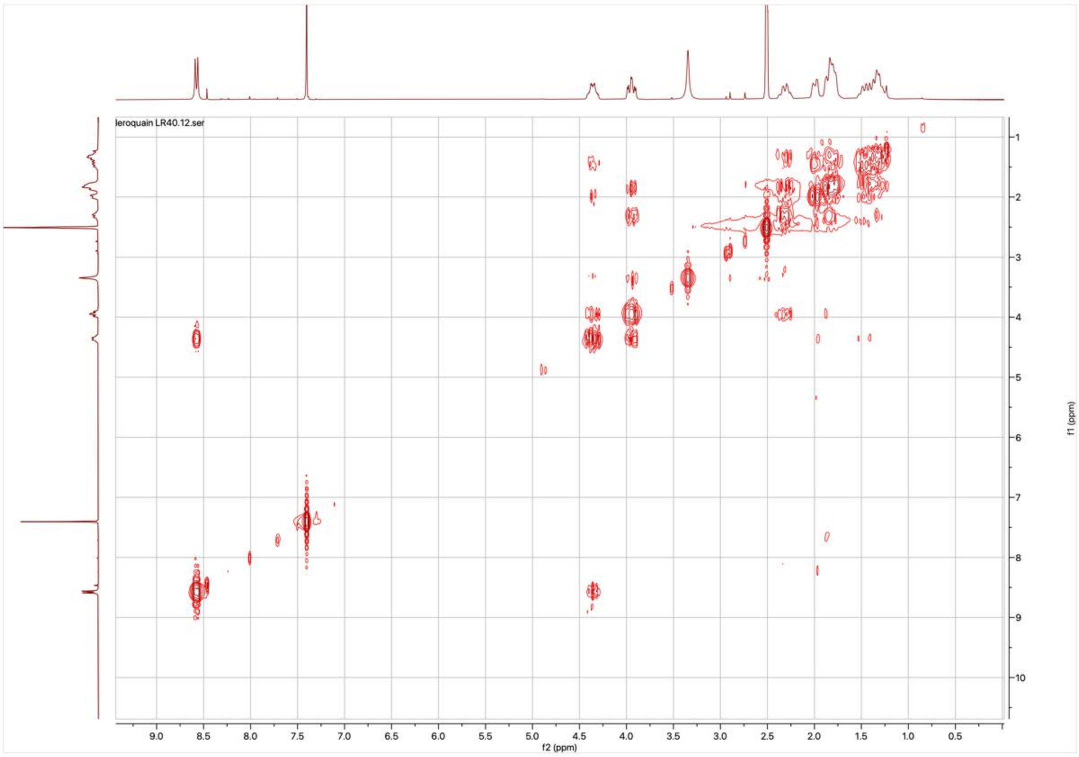

Region of the COESY spectrum of *R*,*R* **O-CyPht$_2$Br$_8$-H** in DMSO-d_6 at room temperature (400 MHz).

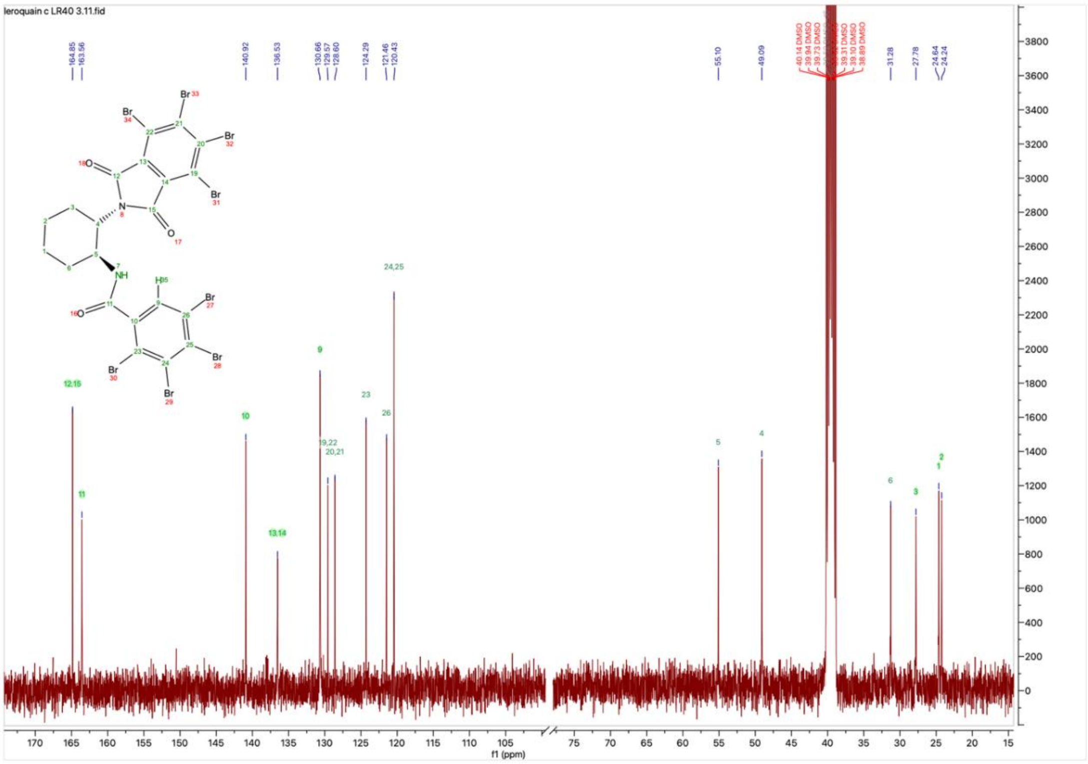

^{13}C NMR spectrum of compound *R*,*R* **O-CyPht$_2$Br$_8$-H** in DMSO-d_6 at room temperature (100 MHz).

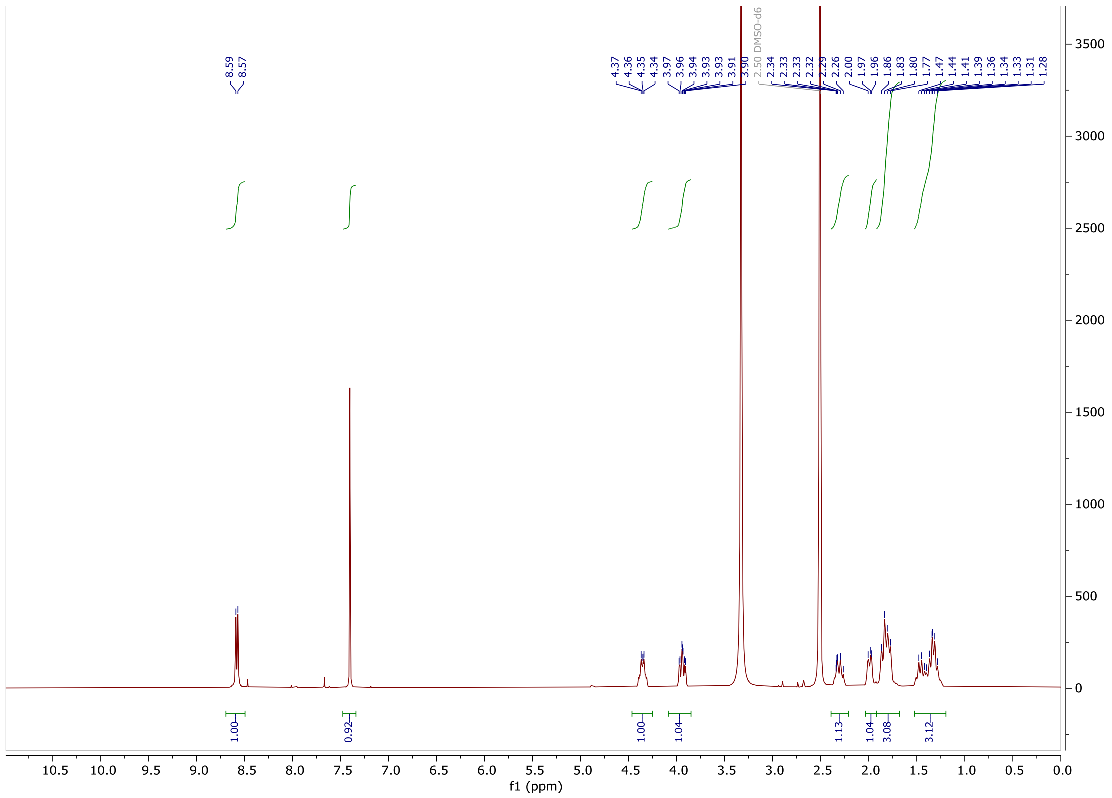

^{1}H NMR spectrum of compound *R*,*R* **O-CyPht$_2$Br$_8$-H** in DMSO-d_6 at room temperature (400 MHz).

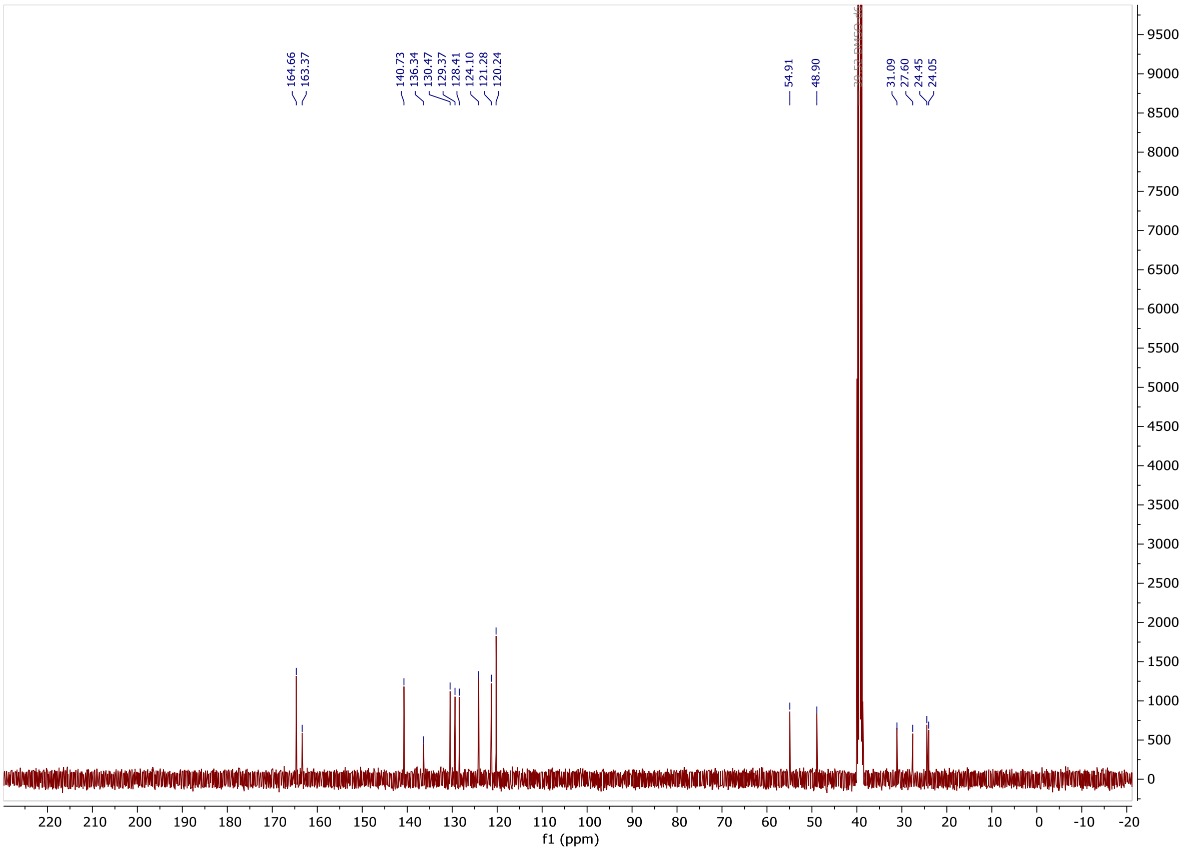

^{13}C NMR spectrum of compound *R*,*R* **O-CyPht$_2$Br$_8$-H** in DMSO-d_6 at room temperature (100 MHz).

H. Theoretical calculations

Methods

The total, transition energies, as well as the geometries and vibrations of all systems have been computed at the (Time-Dependent) Density Functional Theory (TD-DFT) level.

We have selected the PBE0 functional[1] with additional dispersion corrections at the D3-BJ[2] level for obtaining ground-state structures. The geometries were obtained using the 6-31+(d) atomic basis set. The same functional was used to generate global UV/Vis and ECD spectra from the stick transitions determined with TD-DFT, using however a larger atomic basis set, 6-311++G(d,p). To analyze the lowest excited-state we used the CAM-B3LYP[3] functional to avoid spurious CT effects.

All calculations have been performed using the Gaussian16.A03 program,[3] but for the SOC calculations and related S-T gaps that have been achieved with ORCA.[4] In Gaussian used tightened self-consistent field (10^{-10} a.u.) and geometry optimization (10^{-5} a.u.) convergence thresholds, two-electron accuracy limit (10^{-14} a.u.), and a large DFT integration grid (so-called *superfine* grid, a pruned grid).

The ground states were optimized with DFT and the lowest excited singlet with TD-DFT. The nature of the ground-state stationary points was confirmed by analytical Hessian calculations that returned 0 (minima) imaginary vibrational modes. The values reported below are vertical absorption and emission, as obtained by TD-DFT.

The ECD spectra were simulated by convoluting the TD-DFT "stick" contributions with a Gaussian showing a HWHM of 1400 cm^{-1}. For these calcultions, we use the ground-state geometry determined above and the B3LYP functional.

Eventually the spin-orbit coupling elements were computed using the same M06-2X hybrid functional, the ZORA Hamiltonian, the *def2*-TVP basis set and the CPCM(SMD) solvent model for the calculations. TDA was not applied. The reported SOC values reported in the text have been computed as:

$$\sqrt{\frac{1}{3}S_x^2 + \frac{1}{3}S_y^2 + \frac{1}{3}S_z^2}$$

For the sake of consistency, the S-T gaps given in the main text when studying the ISC process have been computed with ORCA and the same methodology.

[1] C. Adamo, V. Barone, *J. Chem. Phys.* **1999**, *110*, 6158–6170.

[2] S. Grimme, S. Ehrlich, L. Goerigk, *J. Comput. Chem.* **2011**, *32*, 1456—1465.

[3] T. Yanai, D. P. Tew, N. C. Handy, *Chem. Phys. Lett.* **2004**, *393*, 51–57.

[3] M. J. Frisch, G. W. Trucks, H. B. Schlegel, G. E. Scuseria, M. A. Robb, J. R. Cheeseman, G. Scalmani, V. Barone, B. Mennucci, G. A. Petersson, H. Nakatsuji, M. Caricato, X. Li, H. P. Hratchian, A. F. Izmaylov, J. Bloino, G. Zheng, J. L. Sonnenberg, M. Hada, M. Ehara, K. Toyota, R. Fukuda, J. Hasegawa,

M. Ishida, T. Nakajima, Y. Honda, O. Kitao, H. Nakai, T. Vreven, J. A. Montgomery, Jr., J. E. Peralta, F. Ogliaro, M. Bearpark, J. J. Heyd, E. Brothers, K. N. Kudin, V. N. Staroverov, R. Kobayashi, J. Normand, K. Raghavachari, A. Rendell, J. C. Burant, S. S. Iyengar, J. Tomasi, M. Cossi, N. Rega, J. M. Millam, M. Klene, J. E. Knox, J. B. Cross, V. Bakken, C. Adamo, J. Jaramillo, R. Gomperts, R. E. Stratmann, O. Yazyev, A. J. Austin, R. Cammi, C. Pomelli, J. W. Ochterski, R. L. Martin, K. Morokuma, V. G. Zakrzewski, G. A. Voth, P. Salvador, J. J. Dannenberg, S. Dapprich, A. D. Daniels, O. Farkas, J. B. Foresman, J. V. Ortiz, J. Cioslowski and D. J. Fox, Gaussian 16 Revision A.03, **2016**, Gaussian Inc. Wallingford CT.

[4] F. Neese, *WIREs Comput. Mol. Sci.* **2018**, *8*, e1327

Additional results

For **C-Cy(PhtBr$_4$)$_2$** only one conformer was found in the ground electronic state (S0). For **O-CyPht$_2$Br$_8$-H** we could identify two possible conformers, whereas a larger series could be obtained for the carboxylic-bearing **O-CyPht$_2$Br$_8$-CO$_2$H**. These structures and their relative free energies, as provided by DFT, are displayed in the Figures below.

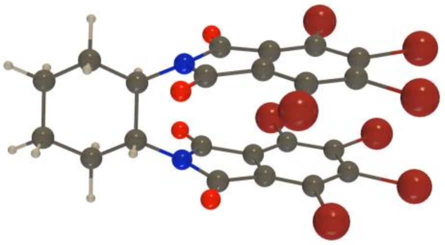

Figure S21. Representation of the optimal structures of **C-Cy(PhtBr$_4$)$_2$**. That structure is stable in the C_2 point group.

Conf. A
(0.0 kcal/mol)

Conf. B
(3.1 kcal/mol)

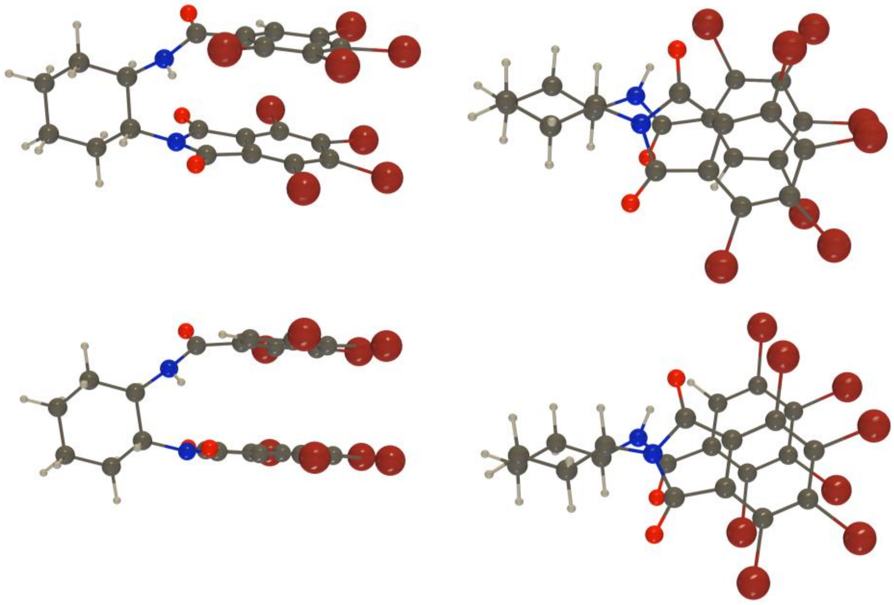

Figure S22. Representation of the optimal structures of **O-CyPht$_2$Br$_8$-H**. We provide the relative free energies on the lhs. PBE0-D3BJ/6-31+G(d) results.

Conf. A
(3.3 kcal/mol)

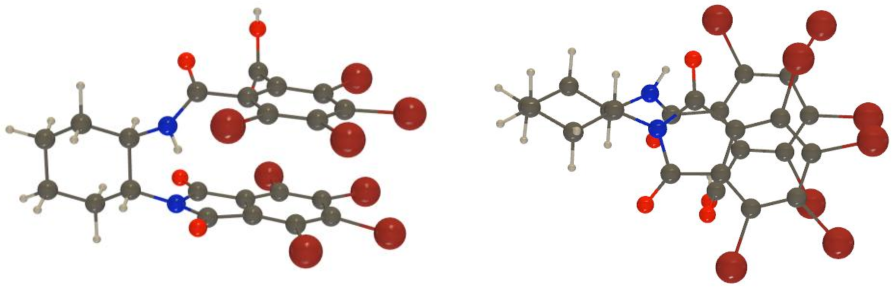

Conf. B
(2.5 kcal/mol)

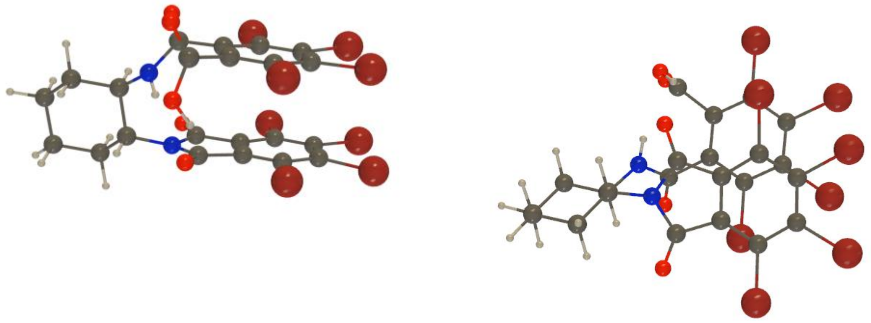

Conf. C
(0.0 kcal/mol)

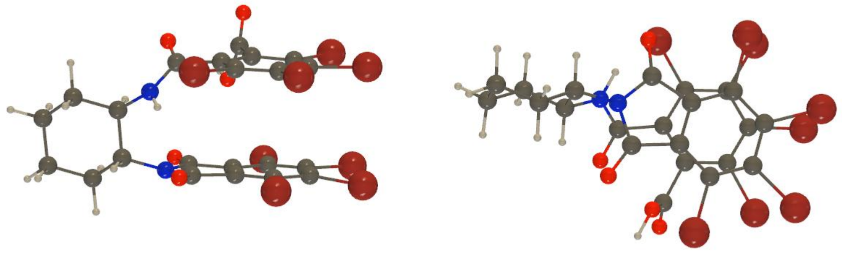

Conf. D
(1.4 kcal/mol)

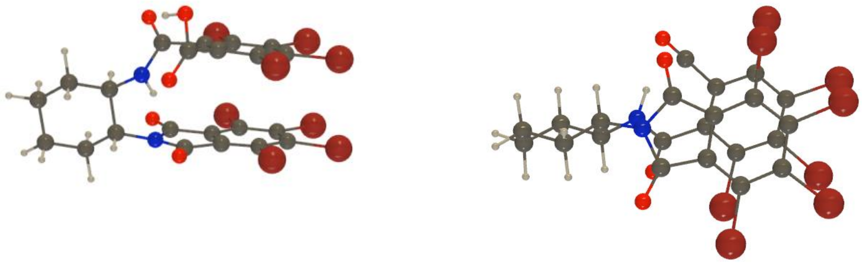

Conf. E
(2.0 kcal/mol)

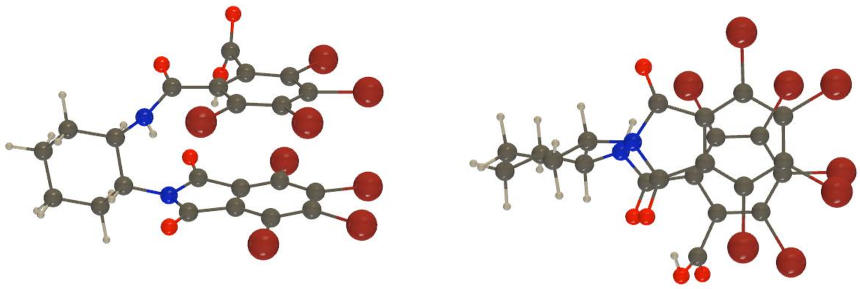

Figure S23. Representation of the optimal structures of **O-CyPht$_2$Br$_8$-CO$_2$H**. We provide the relative free energies on the lhs. PBE0-D3BJ/6-31+G(d) results.

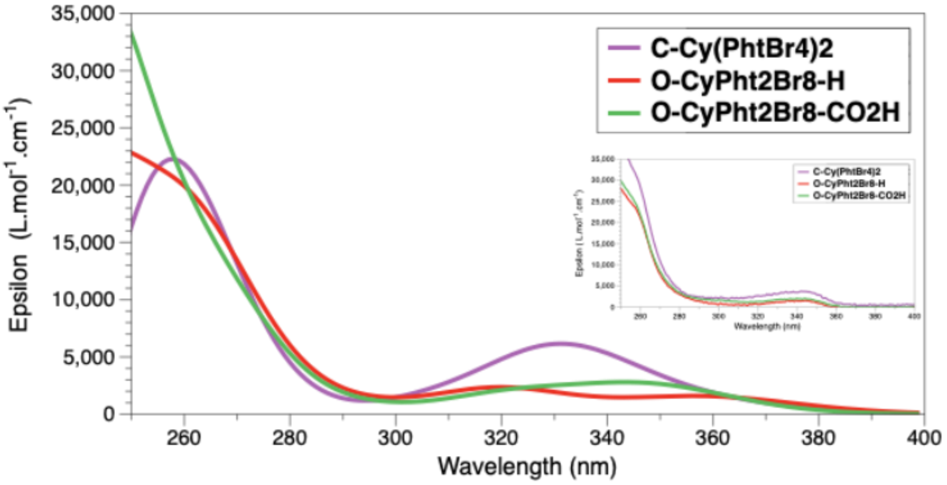

Figure S24.Computed UV/Vis spectra obtained by a convolution of the « stick » PBE0-D3BJ/6-311++G(d,p) transitions for the three compounds. Only the most stable conformer was considered in each case. The inset represents the experimental data (same colour code and scale).

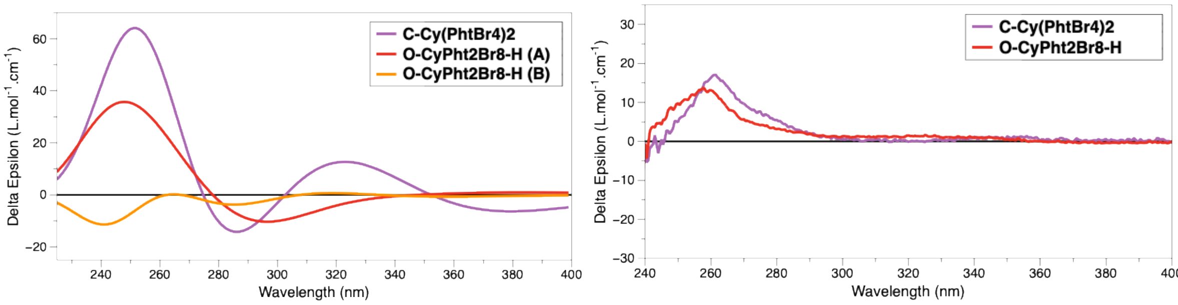

Figure S25. Left: computed ECD spectra obtained by a convolution of the « stick » PBE0-D3BJ/6-311++G(d,p) transitions for two compounds compounds. The ECD spectra of the two conformers are shown for **O-CyPht$_2$Br$_8$-H**. Right: experimental ECD spectra.

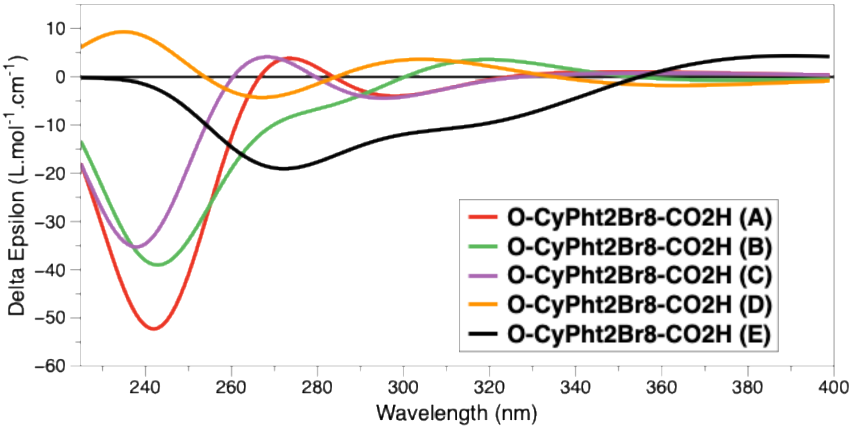

Figure S26.Computed ECD spectra obtained by a convolution of the « stick » PBE0-D3BJ/6-311++G(d,p) transitions for **O-CyPht$_2$Br$_8$-CO$_2$H**. The ECD spectra of the five conformers are shown.

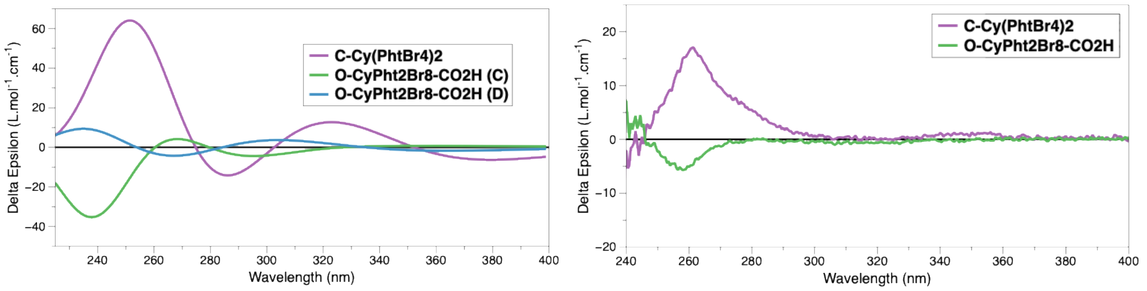

Figure S27. Left: computed ECD spectra obtained by a convolution of the « stick » PBE0-D3BJ/6-311++G(d,p) transitions for two compounds compounds. The ECD spectra of the two most stable conformers are shown for **O-CyPht$_2$Br$_8$- CO$_2$H**. Right: experimental ECD spectra.

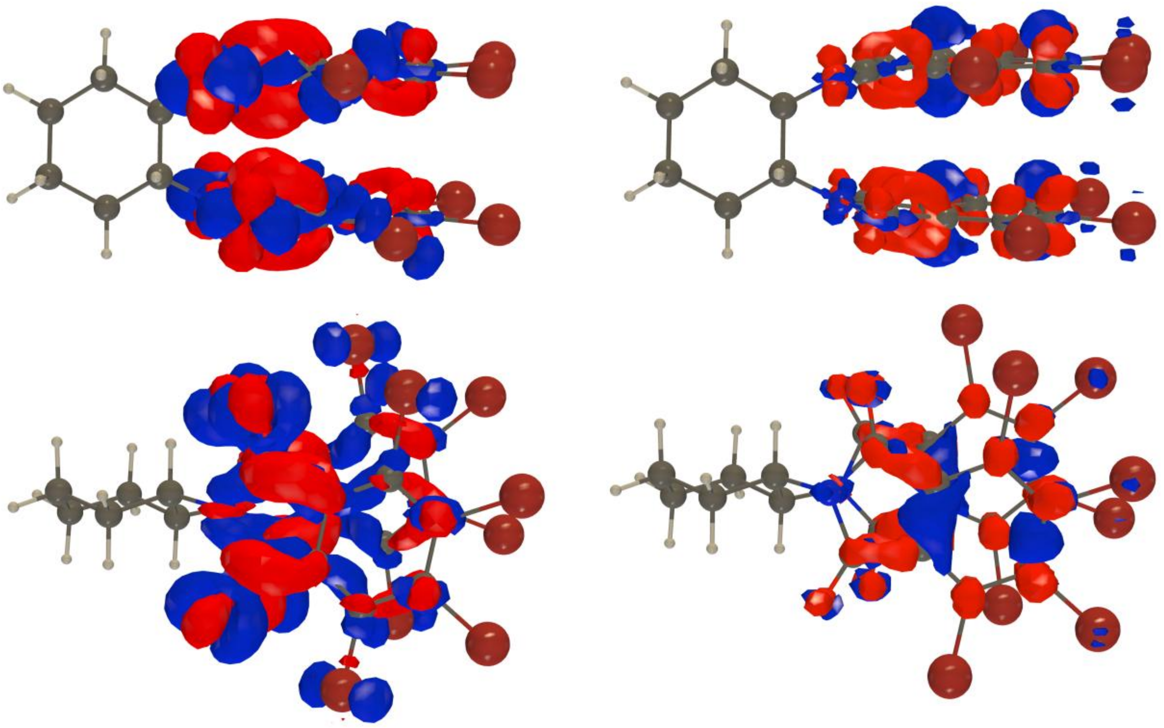

Figure S28. Electron density difference plot of the lowest singlet (left) and triplet (right) excited-states determined at TD-CAM-B3LYP/6-311++G(d,p) level for **C-Cy(PhtBr$_4$)$_2$**. The blue (red) regions indicate decrease (increase) of electron density upon absorption. Controur threshold: 0.001 au.

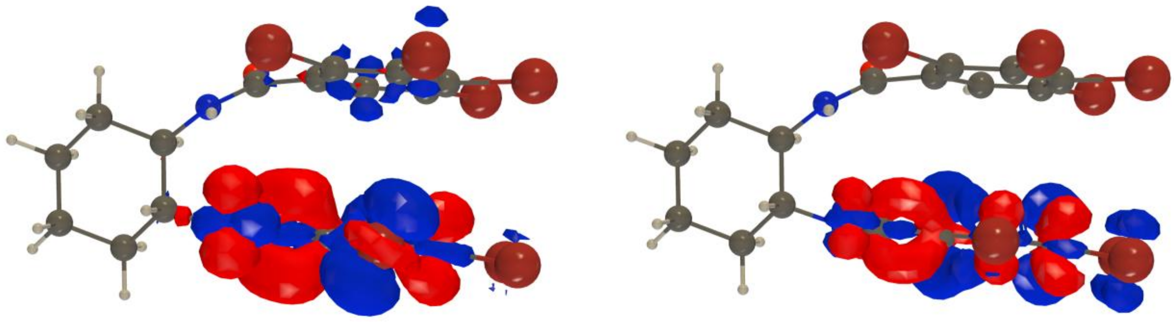

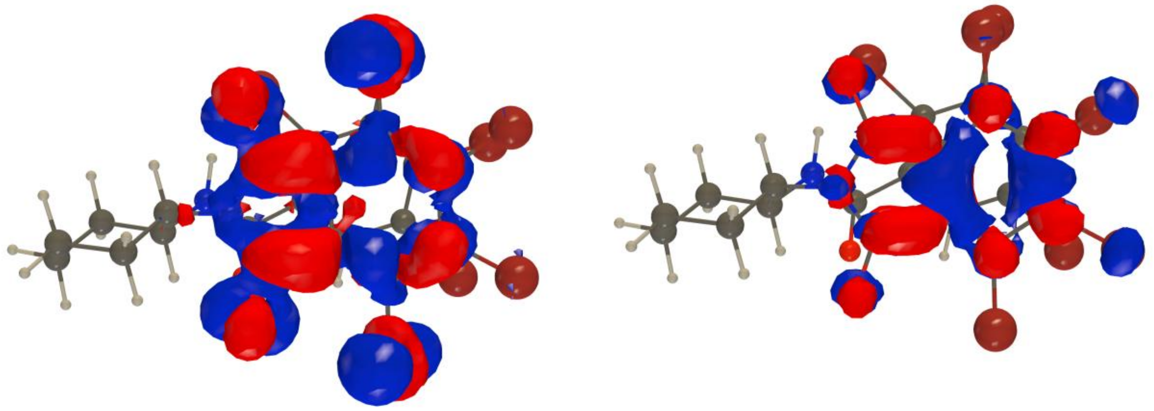

Figure S29. Electron density difference plot of the lowest singlet (left) and triplet (right) excited-states determined at TD-CAM-B3LYP/6-311++G(d,p) level for the most stable conformer of **O-CyPht$_2$Br$_8$-H**.. The blue (red) regions indicate decrease (increase) of electron density upon absorption. Controur threshold: 0.001 au.

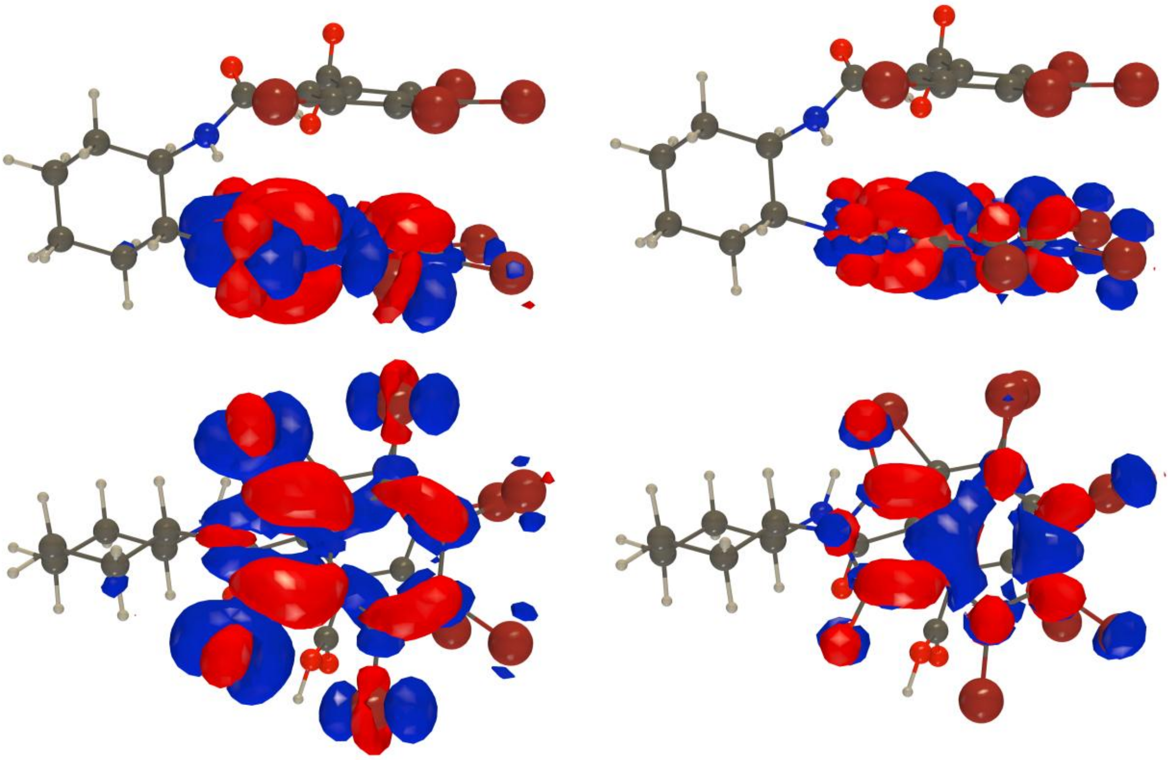

Figure S30. Electron density difference plot of the lowest singlet (left) and triplet (right) excited-states determined at TD-CAM-B3LYP/6-311++G(d,p) level for the most stable conformer of **O-CyPht$_2$Br$_8$-CO$_2$H**. The blue (red) regions indicate decrease (increase) of electron density upon absorption. Controur threshold: 0.001 au.

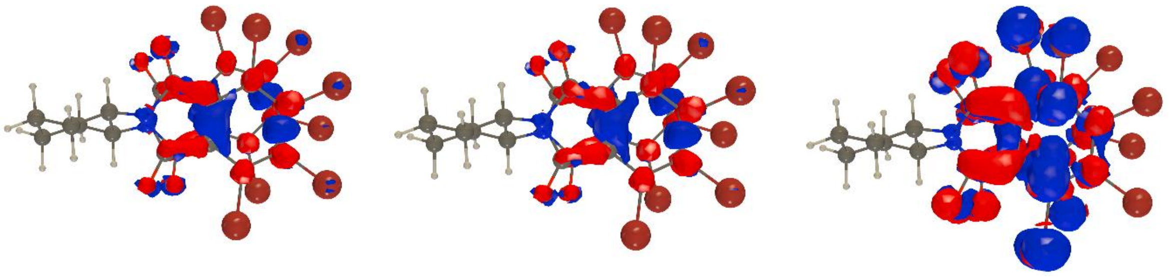

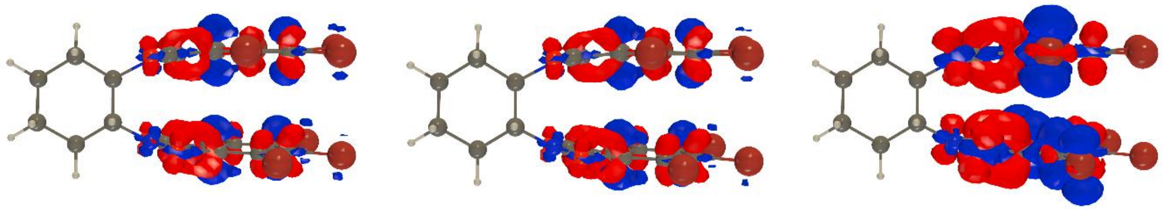

Figure S31. Electron density difference plot of the three lowest triplet excited-states determined at TD-CAM-B3LYP/6-311++G(d,p) level for **C-Cy(PhtBr$_4$)$_2$**. From Left to right: T_1, T_2, T_3. The blue (red) regions indicate decrease (increase) of electron density upon absorption. Controur threshold: 0.001 au.

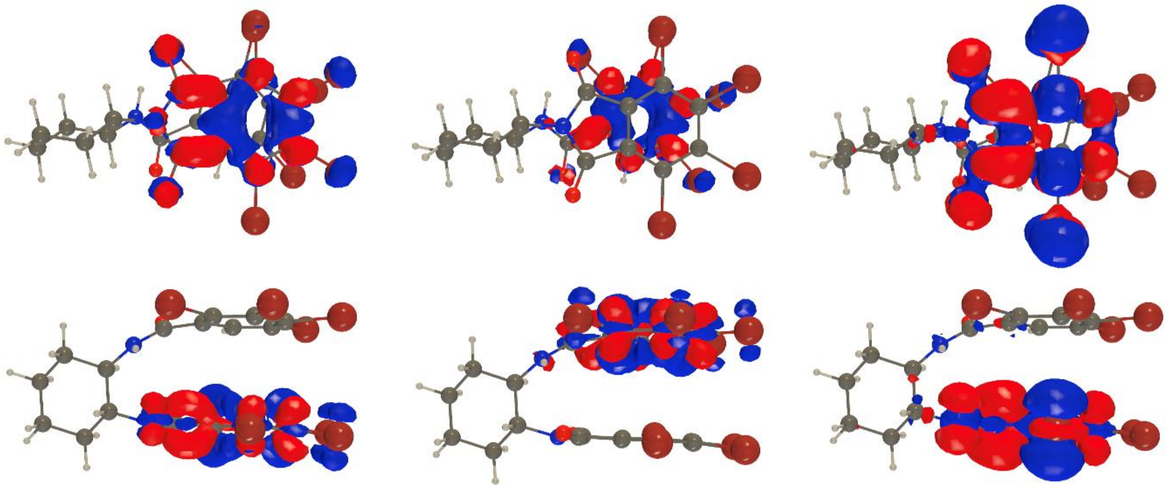

Figure S32. Electron density difference plot of the three lowest triplet excited-states determined at TD-CAM-B3LYP/6-311++G(d,p) level for **O-CyPht$_2$Br$_8$-H**. From Left to right: T_1, T_2, T_3. The blue (red) regions indicate decrease (increase) of electron density upon absorption. Controur threshold: 0.001 au.

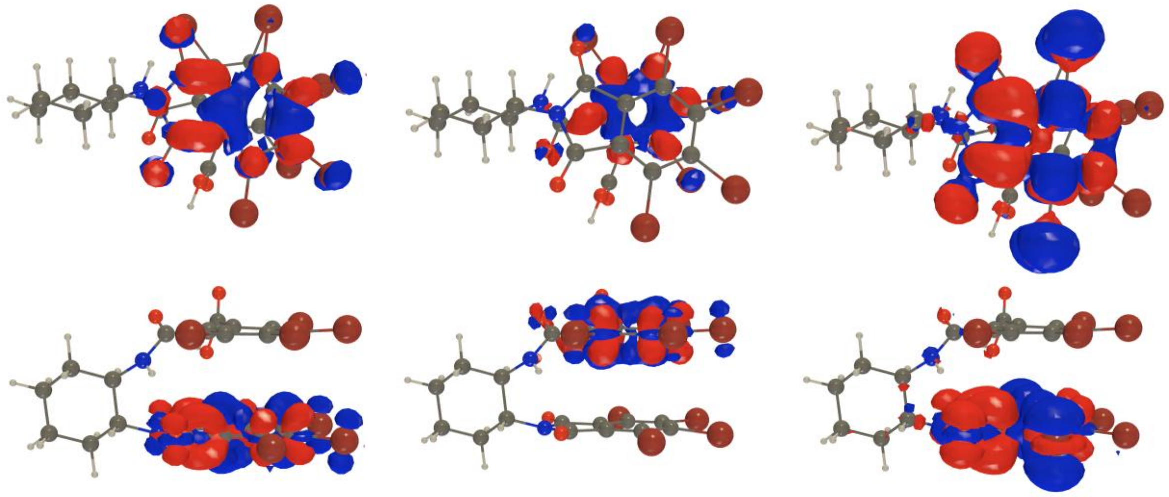

Figure S33. Electron density difference plot of the three lowest triplet excited-states determined at TD-CAM-B3LYP/6-311++G(d,p) level for **O-CyPht$_2$Br$_8$-CO$_2$H**. From Left to right: T_1, T_2, T_3. The blue (red) regions indicate decrease (increase) of electron density upon absorption. Controur threshold: 0.001 au.

Table S2. SOC (cm^{-1}) computed between the lowest S_1 state and the three lowest triplet states on the optimal geometry of the lowest singlet state.
We also report the S-T gaps in eV, a negative value indicating a higher-lying triplet.

		S-T gap	SOC
**C-Cy(PhtBr$_4$)$_2$**	S_1-T_1	0.38	0.1
	S_1-T_2	0.27	1.6
	S_1-T_3	-0.21	6.0
**O-CyPht$_2$Br$_8$-H**	S_1-T_1	0.30	7.6
	S_1-T_2	0.23	47.7
	S_1-T_3	0.05	133.0
**O-CyPht$_2$Br$_8$-CO$_2$H**	S_1-T_1	0.52	6.9
	S_1-T_2	0.37	5.9
	S_1-T_3	0.06	102.5

Geometries

Below are Cartesian coordinates for all key structures (in Å). The most stable conformers are considered.

C-Cy(PhtBr$_4$)$_2$.

S_0 state – PBE0-D3[BJ] optimization, *G*= -21824.612111 au

N	-1.0798280	0.8673880	3.1162640
C	-0.7429200	0.2193550	4.3785660
H	-1.3717600	-0.6782600	4.3972930
C	-1.0389110	1.0595880	5.6150760
H	-2.0939300	1.3567570	5.5997030
H	-0.4386210	1.9767900	5.5855300
C	-0.7216510	0.2512420	6.8726910
H	-0.9090070	0.8638680	7.7623480
H	-1.4053250	-0.6080470	6.9325390
O	-2.5689320	-0.7393860	2.3600280
C	-1.8380390	0.1984020	2.1480820
C	-1.4999260	0.8380810	0.8454900
C	-1.8452790	0.4629440	-0.4396200
C	-1.2409750	1.1559290	-1.5042490
O	0.3227900	2.7103370	3.1918160
C	-0.3659670	1.9390810	2.5688400
C	-0.6051240	1.8761820	1.0935160
C	-0.0015840	2.5706300	0.0608810
C	-0.3227900	2.1897010	-1.2567830
N	1.0798280	-0.8673880	3.1162640
C	0.7429200	-0.2193550	4.3785660
H	1.3717600	0.6782600	4.3972930
C	1.0389110	-1.0595880	5.6150760
H	2.0939300	-1.3567570	5.5997030
H	0.4386210	-1.9767900	5.5855300
C	0.7216510	-0.2512420	6.8726910
H	0.9090070	-0.8638680	7.7623480
H	1.4053250	0.6080470	6.9325390
O	2.5689320	0.7393860	2.3600280
C	1.8380390	-0.1984020	2.1480820
C	1.4999260	-0.8380810	0.8454900
C	1.8452790	-0.4629440	-0.4396200
C	1.2409750	-1.1559290	-1.5042490
O	-0.3227900	-2.7103370	3.1918160
C	0.3659670	-1.9390810	2.5688400
C	0.6051240	-1.8761820	1.0935160
C	0.0015840	-2.5706300	0.0608810
C	0.3227900	-2.1897010	-1.2567830
Br	-1.1693140	-3.9713390	0.4187730
Br	-0.4681370	-3.0948490	-2.6778380
Br	1.7307530	-0.7413690	-3.2535930
Br	3.1108940	0.8714330	-0.7387980
Br	-3.1108940	-0.8714330	-0.7387980
Br	-1.7307530	0.7413690	-3.2535930
Br	0.4681370	3.0948490	-2.6778380
Br	1.1693140	3.9713390	0.4187730

S_1 state – TD-CAM-B3LYP optimization, *G*= -21828.660011 au

N	-3.1699420	-0.2289830	-1.3580340
C	-4.4584190	-0.3623000	-0.6836060
H	-4.5416940	-1.4380550	-0.5059590
C	-5.6727960	0.0908800	-1.4864690
H	-5.6635320	-0.3994100	-2.4654090
H	-5.6191310	1.1718800	-1.6587660
C	-6.9443020	-0.2797840	-0.7137760
H	-7.8267000	0.0780640	-1.2551570
H	-7.0244440	-1.3744920	-0.6719890
O	-2.7223860	-2.5084600	-1.4112860
C	-2.3424560	-1.3568920	-1.4522400

C	-0.9712360	-0.8312000	-1.5735720
C	0.2444700	-1.5189820	-1.5915010
C	1.4376870	-0.7829340	-1.6419950
O	-2.9413950	2.0796910	-1.5350270
C	-2.4533190	0.9678340	-1.5071320
C	-1.0254930	0.5613630	-1.6043670
C	0.1594100	1.3024620	-1.6846200
C	1.3955930	0.6277220	-1.6999180
N	-3.1699410	0.2289830	1.3580340
C	-4.4584190	0.3623000	0.6836060
H	-4.5416940	1.4380550	0.5059590
C	-5.6727950	-0.0908800	1.4864700
H	-5.6635310	0.3994100	2.4654100
H	-5.6191300	-1.1718800	1.6587670
C	-6.9443020	0.2797840	0.7137770
H	-7.8267000	-0.0780640	1.2551580
H	-7.0244440	1.3744920	0.6719900
O	-2.7223860	2.5084610	1.4112860
C	-2.3424550	1.3568920	1.4522400
C	-0.9712360	0.8312000	1.5735720
C	0.2444700	1.5189820	1.5915010
C	1.4376880	0.7829340	1.6419950
O	-2.9413950	-2.0796910	1.5350270
C	-2.4533190	-0.9678340	1.5071320
C	-1.0254930	-0.5613630	1.6043670
C	0.1594100	-1.3024620	1.6846200
C	1.3955930	-0.6277220	1.6999180
Br	0.0491390	-3.1442790	1.8822090
Br	2.9775430	-1.5950900	1.9516610
Br	3.0740410	1.6791790	1.7913410
Br	0.2410060	3.3720100	1.6541390
Br	0.2410050	-3.3720100	-1.6541390
Br	3.0740400	-1.6791790	-1.7913410
Br	2.9775430	1.5950900	-1.9516610
Br	0.0491390	3.1442790	-1.8822090

O-CyPht$_2$Br$_8$-H.

S_0 state – PBE0-D3^{BJ} optimization, *G*= -21712.566796 au

N	3.0683960	0.9590480	1.1705960
C	4.4543750	0.6303480	0.8653620
H	4.6422920	-0.2836670	1.4441280
C	5.4625320	1.6911610	1.2889620
H	5.3355090	1.9086560	2.3561050
H	5.2686820	2.6191800	0.7391610
C	6.8769750	1.1857730	1.0036420
H	7.6060610	1.9592250	1.2722180
H	7.0886340	0.3189770	1.6473020
O	2.5686270	-1.0835410	2.1365240
C	2.2141890	-0.0142380	1.6911340
C	0.8320350	0.5108700	1.5275680
C	-0.3821270	-0.0450570	1.8856740
C	-1.5454830	0.6917200	1.5944630
O	2.8455280	2.9413150	-0.0103340
C	2.3576980	2.0115730	0.5868030
C	0.9141480	1.7206570	0.8442550
C	-0.2213670	2.4235590	0.4889330
C	-1.4651160	1.9149250	0.9087460
N	3.5368920	-0.6116220	-1.0103080
C	4.6043410	0.2927290	-0.6316760
H	4.4558410	1.2018160	-1.2240400
C	6.0057560	-0.2458610	-0.8934130
H	6.1021440	-0.4820080	-1.9587940
H	6.1458920	-1.1858100	-0.3389930
C	7.0510590	0.7814690	-0.4603410
H	8.0590810	0.3826960	-0.6249690
H	6.9586650	1.6735020	-1.0963310
O	2.8098020	0.6421460	-2.7477070
C	2.5977240	-0.2244950	-1.9107660

C	1.1871970	-0.7514980	-1.7407530
C	0.7475560	-1.9449400	-1.1562270
C	-0.6230690	-2.1782330	-0.9720950
C	0.2310190	0.1746970	-2.1540090
C	-1.1236720	-0.0457720	-1.9794050
C	-1.5687950	-1.2275230	-1.3830980
Br	-0.4496950	-1.6513550	2.8253510
Br	-3.1949600	0.0770130	2.2069240
Br	-3.0044540	2.9195300	0.6032340
Br	-0.0941840	3.9978880	-0.4942100
Br	1.9589620	-3.3118700	-0.7045940
Br	-1.2077420	-3.8035700	-0.2582040
Br	-3.3951260	-1.5776990	-1.2588040
Br	-2.3050750	1.2249660	-2.6851660
H	3.3124190	-1.3600900	-0.3669100
H	0.5837670	1.0833950	-2.6299130

S_1 *state* – TD-CAM-B3LYP optimization, *G*= -21716.540603 au

N	3.2666950	-0.0575310	1.3058710
C	4.5954850	-0.0562190	0.6948410
H	4.8294990	-1.1200290	0.5878260
C	5.6781540	0.5957130	1.5480920
H	5.6923250	0.1274660	2.5375280
H	5.4416540	1.6575600	1.6922310
C	7.0362930	0.4423740	0.8549230
H	7.8092080	0.9512200	1.4404110
H	7.3077540	-0.6219660	0.8368830
O	2.9859410	-2.3663570	1.2353060
C	2.4865690	-1.2595130	1.3580630
C	1.1066300	-0.8248040	1.5198330
C	-0.1031800	-1.5068170	1.6184670
C	-1.2847980	-0.7756280	1.7213910
O	2.8256340	2.2532050	1.2967120
C	2.4320370	1.0425560	1.3758640
C	1.1066670	0.5788120	1.5144070
C	-0.0689850	1.3195700	1.5818190
C	-1.2644060	0.6500810	1.7050060
N	3.3877600	0.0854690	-1.4209390
C	4.5502000	0.5871700	-0.7053180
H	4.3937480	1.6649640	-0.6009380
C	5.8863950	0.3686410	-1.4092380
H	5.8411560	0.8229630	-2.4034180
H	6.0559370	-0.7075920	-1.5497930
C	7.0163040	0.9840050	-0.5780730
H	7.9811750	0.7991340	-1.0620170
H	6.8829290	2.0738620	-0.5522670
O	2.7823230	2.1017460	-2.2207890
C	2.4748430	0.9554580	-1.9236680
C	1.0035430	0.5661360	-1.9447790
C	0.4041850	-0.6963270	-1.8645400
C	-0.9855820	-0.8163340	-1.7411220
C	0.1764850	1.6850830	-1.9072690
C	-1.1915550	1.5781350	-1.7542890
C	-1.7919450	0.3248960	-1.6766660
Br	-0.0794660	-3.3759820	1.6446410
Br	-2.9180540	-1.6703670	1.9334290
Br	-2.8413940	1.6372240	1.9209270
Br	0.1461140	3.1785780	1.5468000
Br	1.4169920	-2.2842250	-2.0272530
Br	-1.8008310	-2.5083390	-1.7535570
Br	-3.6540150	0.1900420	-1.5567330
Br	-2.1807950	3.1760050	-1.6884910
H	3.1068480	-0.8697810	-1.2500660
H	0.6461340	2.6571780	-1.9789300

O-CyPht$_2$Br$_8$-CO$_2$H.

S_0 state – PBE0-D3^{BJ} optimization, *G*= -21900.936558 au.

N	3.0465720	-0.8406430	-1.3757000
C	4.4139030	-0.4322230	-1.0741760
H	4.5638420	0.4628790	-1.6920940
C	5.4787480	-1.4572530	-1.4423550
H	5.3719920	-1.7267120	-2.4997900
H	5.3286810	-2.3705330	-0.8565670
C	6.8629660	-0.8688110	-1.1687570
H	7.6318440	-1.6150280	-1.4007770
H	7.0373660	-0.0186830	-1.8449750
O	2.4210860	1.2171690	-2.2271610
C	2.1314480	0.1088100	-1.8282800
C	0.7830350	-0.4855280	-1.6548450
C	-0.4610260	0.0447700	-1.9432810
C	-1.5858740	-0.7399050	-1.6334940
O	2.9509870	-2.9139710	-0.3384340
C	2.4038980	-1.9712440	-0.8582950
C	0.9407460	-1.7388870	-1.0706110
C	-0.1546310	-2.5184520	-0.7496790
C	-1.4336800	-1.9983960	-1.0252770
N	3.4201690	0.8655760	0.7176980
C	4.5307280	-0.0138720	0.4045740
H	4.4118340	-0.8977860	1.0421170
C	5.9054110	0.5958300	0.6573490
H	5.9762060	0.8818050	1.7123300
H	6.0068080	1.5163430	0.0638260
C	7.0018150	-0.3986760	0.2790370
H	7.9879060	0.0535820	0.4375850
H	6.9442740	-1.2677500	0.9501390
O	2.9587880	-0.0530970	2.7307160
C	2.6060240	0.6156690	1.7696950
C	1.1511370	1.0120040	1.6341960
C	0.6466930	2.1567410	1.0077070
C	-0.7324920	2.3357200	0.8473740
C	0.2447100	0.0639670	2.1307780
C	-1.1256440	0.2094020	1.9253960
C	-1.6233850	1.3541300	1.2974550
Br	-0.6139800	1.6813520	-2.8213200
Br	-3.2770410	-0.1516800	-2.1517530
Br	-2.9356850	-3.0422210	-0.6682360
Br	0.0604050	-4.2337890	-0.0536050
Br	1.8024580	3.5339760	0.4570040
Br	-1.3923130	3.9219730	0.1156640
Br	-3.4638980	1.6297030	1.1826920
Br	-2.2924320	-1.0969560	2.5881650
H	3.1123050	1.5048840	-0.0039770
C	0.7134850	-1.1383810	2.9144950
O	0.5933130	-1.2520700	4.1052060
O	1.1566990	-2.0915910	2.0871520
H	1.4616950	-2.8441210	2.6229980

S_1 state – TD-CAM-B3LYP optimization, *G*= -21905.037432 au

N	-3.0001900	0.0181540	-1.5707210
C	-4.4036250	-0.1591230	-1.2195520
H	-4.5925690	-1.2192200	-1.4194150
C	-5.3883930	0.6697560	-2.0376550
H	-5.2220450	0.4795470	-3.1033560
H	-5.2039520	1.7341120	-1.8653950
C	-6.8185220	0.2926390	-1.6378240
H	-7.5327310	0.9113990	-2.1918160
H	-7.0132460	-0.7486410	-1.9303920
O	-2.5478670	-2.2644010	-1.4812190
C	-2.1544370	-1.0971000	-1.5715210
C	-0.8055380	-0.5670610	-1.5821760
C	0.4410540	-1.2237060	-1.6683000
C	1.6156490	-0.4767770	-1.6777660
O	-2.8071580	2.3385990	-1.4300970

C	-2.2900060	1.2309310	-1.4989820
C	-0.8784510	0.8402200	-1.5207120
C	0.3059550	1.5915870	-1.4892520
C	1.5494630	0.9420570	-1.5709710
N	-3.5379960	-0.6016210	1.0081590
C	-4.6141250	0.0832220	0.2885280
H	-4.4925610	1.1497170	0.4993480
C	-6.0221050	-0.3397220	0.6899900
H	-6.1593440	-0.1632320	1.7612370
H	-6.1396320	-1.4184540	0.5187820
C	-7.0509600	0.4443580	-0.1308100
H	-8.0636920	0.1181050	0.1297490
H	-6.9848570	1.5069510	0.1394920
O	-3.1947360	1.0430290	2.5123810
C	-2.7592600	0.1108180	1.8530620
C	-1.2520060	-0.0977670	1.8249960
C	-0.5600430	-1.2981880	1.6053280
C	0.8543610	-1.3161780	1.5455320
C	-0.5152440	1.0773370	1.9474050
C	0.8804410	1.0767120	1.7754750
C	1.5702710	-0.1244470	1.6145830
Br	0.4473830	-3.0637340	-1.8917030
Br	3.2635860	-1.3298990	-1.9321460
Br	3.1232090	1.9452330	-1.6418200
Br	0.1925360	3.4403980	-1.4747400
Br	-1.4766900	-2.9243560	1.5689180
Br	1.7690280	-2.9416470	1.4843690
Br	3.4414380	-0.1301820	1.6293440
Br	1.7940480	2.6992410	1.8794320
H	-3.1482840	-1.4118610	0.5441930
C	-1.1547060	2.3858630	2.3574020
O	-1.0688960	2.8176700	3.4764000
O	-1.6681000	3.0275390	1.3094570
H	-2.0931600	3.8469180	1.6215720

I. Transient absorption study

Methods for transient absorption studies:

Transient absorption studies were performed using a commercial fsTA setup (HARPIA-TA, Light Conversion) that utlises a 1030 nm Yb:KGW 25 kHz laser source (Pharos, Light Conversion). Pump excitation was generated using a non-linear optical parametric amplifier (OPA) (Orpheus + Lyra, Light Conversion). UV-VIS (380-490 nm) probe pulses were generated through irradiation of a sapphire crystal with 515 nm radiation. Temporal resolution is obtained by a delay stage to maximum time of 8 ns after laser excitation. Global analysis of the data was performed to obtain decay associated spectra (DAS) that reflect spectral changes in the TA for fitted time constants. The DAS and time constants were convoluted with an instrument response function (σ) to obtain best fits of the data as described in Ref. 3.[3]

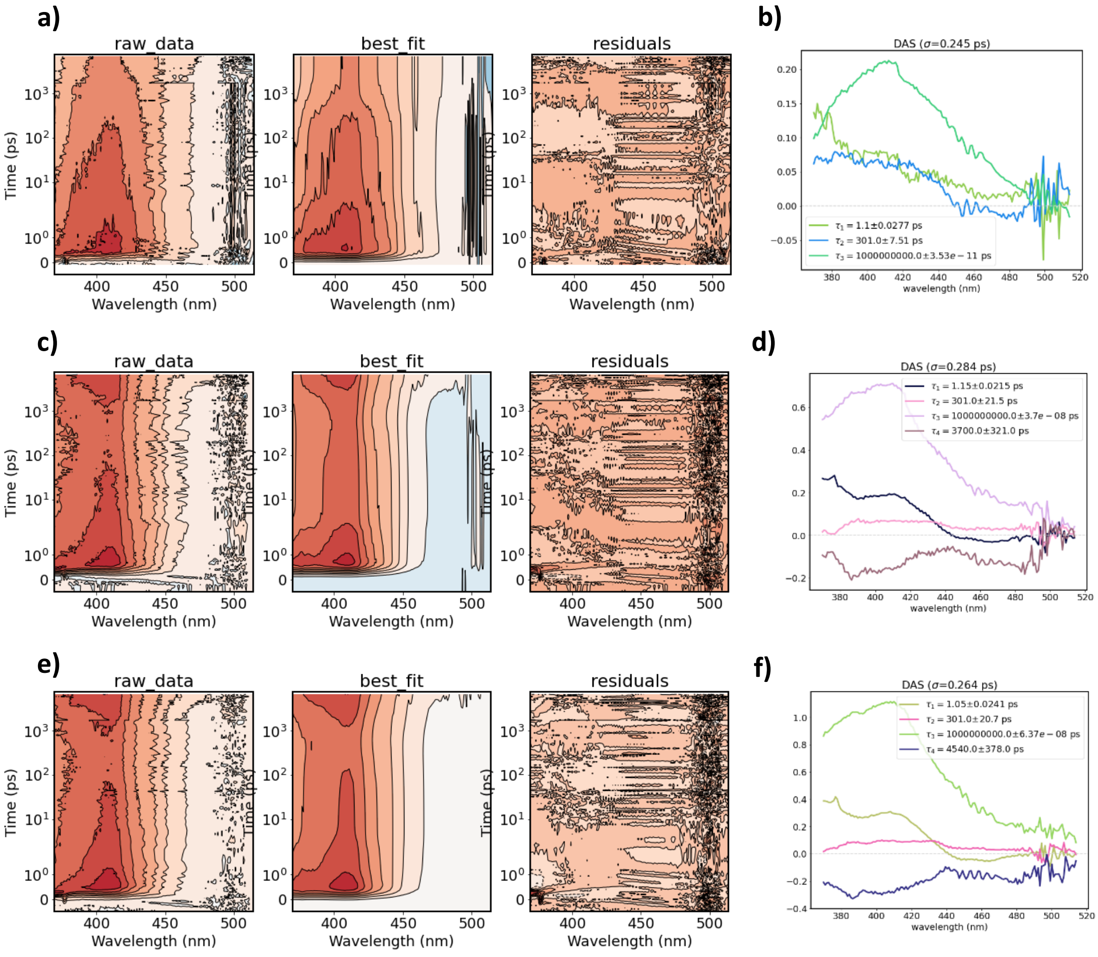

Figure S34. Transient absorption (TA) data and global analysis for **$CyPhtBr_4$** in chloroform at different concentrations: **a)** 20 µM, **c)** 100 µM, **e)** 1 mM. The samples were photoexcited at 343 nm (0.46 µJ per pulse at 5 kHz). The 'raw_data' are indicated as contour plots. The 'best_fit' and 'residuals' plots from global analysis are shown. Global analysis was performed using minimal three and four decay constants (τ) as shown for the corresponding decay associated spectra (DAS) from samples at different concentrations: **b)** 20 µM, **d)** 100 µM, **e)** 1 mM.

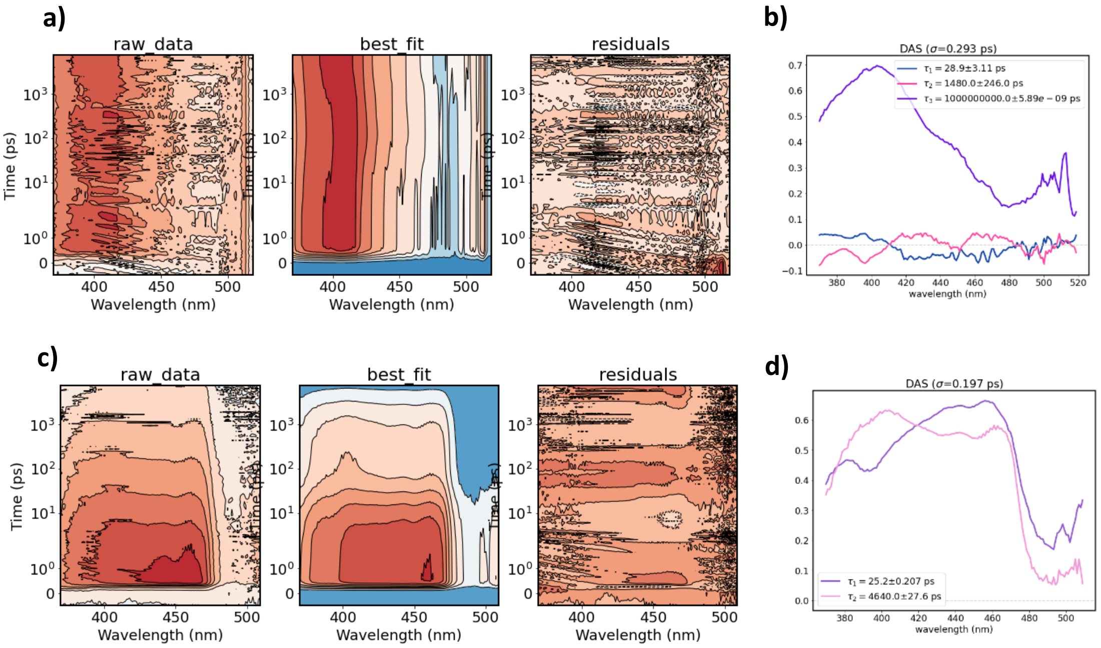

Figure S35. Transient absorption (TA) data and global analysis for **C-Cy(PhtBr$_4$)$_2$** in **a)** chloroform at 1 mM concentration and **c)** 5% wt. doped in PMMA polymer films. The samples were photoexcited at 340 nm (0.48 µJ per pulse at 5 kHz). The 'raw_data' are indicated as contour plots. The 'best_fit' and 'residuals' plots from global analysis are shown. Global analysis was performed using minimal two and three decay constants (τ) as shown for the corresponding decay associated spectra (DAS) in: **b)** solution and **d)** polymer films.

References

[1] L. Favereau, C. Quinton, C. Poriel, T. Roisnel, D. Jacquemin, J. Crassous, *J. Phys. Chem. Lett.* **2020**, *11*, 6426.
[2] C. Demangeat, M. Remond, C. Quinton, T. Roisnel, L. Favereau, *Chem. Eur. J.* **2024**, *30*, e202401506.
[3] I. H. M. van Stokkum, et al., *Biochim. Biophys. Acta - Bioenergetics*, **2004**, *1657*, 82.
